# Complete deconvolution of cellular mixtures based on linearity of transcriptional signatures

**DOI:** 10.1038/s41467-019-09990-5

**Published:** 2019-05-17

**Authors:** Konstantin Zaitsev, Monika Bambouskova, Amanda Swain, Maxim N. Artyomov

**Affiliations:** 1 Department of Pathology and Immunology, Washington University School of Medicine, Saint Louis, MO 63110 USA; 2 Computer Technologies Department, ITMO University, Saint Petersburg, 197101 Russia; 3 Present Address: ITMO University Computer Technologies Department, 49 Kronverkskiy Prospekt, Saint-Petersburg, 197101 Russia; 4 Present Address: Washington University Department of Pathology & Immunology, 660S Euclid Ave, St. Louis, MO 63110 USA

**Keywords:** RNA sequencing, Data processing, Statistical methods

## Abstract

Changes in bulk transcriptional profiles of heterogeneous samples often reflect changes in proportions of individual cell types. Several robust techniques have been developed to dissect the composition of such mixed samples given transcriptional signatures of the pure components or their proportions. These approaches are insufficient, however, in situations when no information about individual mixture components is available. This problem is known as the  complete deconvolution problem, where the composition is revealed without any a priori knowledge about cell types and their proportions. Here, we identify a previously unrecognized property of tissue-specific genes – their mutual linearity – and use it to reveal the structure of the topological space of mixed transcriptional profiles and provide a noise-robust approach to the complete deconvolution problem. Furthermore, our analysis reveals systematic bias of all deconvolution techniques due to differences in cell size or RNA-content, and we demonstrate how to address this bias at the experimental design level.

## Abstract (web summary)

Complete gene expression deconvolution remains a challenging problem. Here, the authors provide a solution based on the recognition that expression levels of cell type specific genes are mutually linear across mixtures and mutually linear gene clusters correspond to cell type-specific signatures.

## Introduction

There are over 200 distinct cell types in the human body^1,2^, and many more subtypes are discovered regularly due to advances in cell sorting, imaging, and single-cell profiling technologies. However, for many complex biological mixtures, exhaustive knowledge of individual cell types and their specific markers is lacking. Yet, such complex tissue samples are routinely collected and profiled during clinical practice and biological research providing a tremendous yet underused biomedical resource.

This complexity has been tackled computationally, resulting in a group of approaches referred to as expression deconvolution methods^3–11^. The general premise of these deconvolution methods assumes that expression signals from each cell type are linearly additive, making the contribution of each cell type proportional to its fraction in the mixture. The existing partial deconvolution methods rely on marker genes, i.e. genes that are known to be expressed in a cell-specific manner^3,4,12^. Current state-of-the-art methods either fit their algorithms to a specific platform and tissue type (e.g. blood/Cibersort^5^, PERT^6^, or tumor/TIMER^13^, DeMix^14^) or use an iterative approach to refine an initial list of marker genes and improve algorithm convergence^12^. At present, deconvolution based on cell-specific markers can be performed quite robustly in the appropriate context. However, in circumstances when little to no information about the underlying cell types is available, current deconvolution methods can be quite unstable^7^.

Here, we propose a strategy to perform complete deconvolution of transcriptional profiles that is robust to technical and biological noise and can reveal the subpopulation structure of complex mixtures without any a priori knowledge about the underlying cell types. To achieve this, we introduce the notion of mutual linearity of tissue-specific genes and reveal a linear subspace (simplex) generated by changes in cell type frequencies within the cohort of samples. We provide a computational approach to unbiasedly select collinear genes and show that filtering out non-collinear genes dramatically improves the performance of deconvolution approaches in realistic noisy data. We illustrate the power of the approach by applying our method to simulated data with and without noise, published benchmark datasets, human and mouse blood profiling in different platforms, as well as TCGA data.

Furthermore, understanding the linear structure of the space revealed a major underappreciated aspect of both partial and complete deconvolution approaches: individual cell types often have varying cell size (per cell RNA content) which leads to a limitation in identifying cellular frequencies in the mixture. Specifically, it implies that any computational deconvolution of transcriptional data can only accurately deconvolve the fraction of RNA contributed by each cell type, which is not identical to the fraction of specific cells in the mixture. We validate this observation by profiling a collection of mixtures of two cell types of drastically different sizes—HEK cells and Jurkat cells. We show that while one can readily identify specific cell types within the mixture, accurate deconvolution of cellular fractions is only possible when taking into account a relative cell size coefficient that can be derived by using ERCC spike-ins.

## Results

### Cell-type-specific genes are defined by *mutual linearity*

**Fig. 1:**
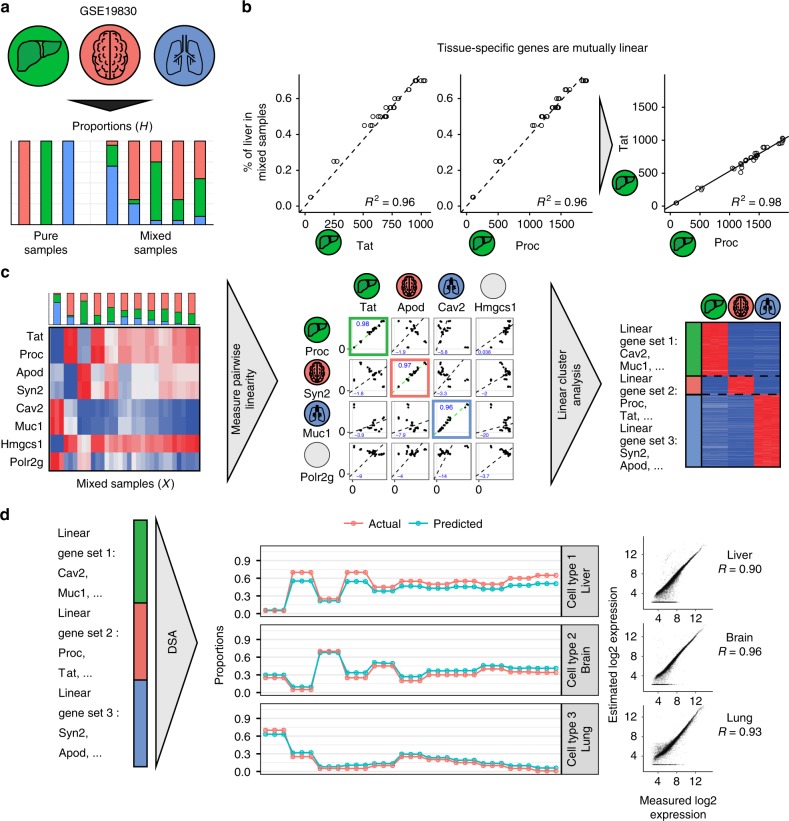
Mutual linearity of cell-type-specific genes enables complete deconvolution. **a** Design of gene expression dataset GSE19830. RNA from three different rat tissues: kidney, brain, and liver were mixed together in known proportions. Gene expression data for pure and mixed samples were measured via microarray profiling. **b** Linear regression without intercept between % of liver and Tat/Proc expression (left), linear regression between Tat and Proc (right). *R*² is coefficient of determination. **c** Approach schematics: given a gene expression matrix of mixed samples as an input we then measured pairwise similarities for each pair of genes and then identified clusters of mutually linear genes. Heatmap on the left shows expression of identified genes in pure tissues from 0 (blue) to gene max (red). **d** Left: Identified gene sets used as DSA algorithm input. Middle: estimated and actual fractions. Right: comparison of estimated and actual gene expression signatures. *R* is Pearson correlation

Cell-type-specific genes are defined by their exclusive expression in only one component within a mixture. In an ideal scenario, expression of a cell-type-specific gene behaves exactly linearly with the proportions of the corresponding mixture component. For instance, the liver-specific genes Tat and Proc are linear with the liver fraction in the mixtures profiled in GSE19830 (ref. ^15^) (Fig. 1a, b). As a consequence, expression levels of the genes specific to the same mixture component are also mutually linear with each other (i.e. obeying equation **y** = *k*·**x**), as shown for Tat and Proc in Fig. 1b (right panel). Importantly, to establish such mutual linearity, one does not need to know the proportions in the mixed samples—only the gene expression profiles of mixed samples are required to evaluate the mutual linearity of each pair of genes.

Mathematically, mutual linearity provides us with a unique measure that can potentially evaluate the cell-type specificity of a gene. Indeed, given an expression profile of all mixed samples, one can directly probe linearity of all pairs of genes, yielding well-defined clusters of genes that are mutually linear to each other (Fig. 1c, left and central panels). Using this approach on known mixtures of lung, liver, and brain tissues (GSE19830) shows that such mutually linear gene clusters directly correspond to tissue-specific gene signatures (Fig. 1c right panel, Supplementary Data 1). The mutually linear gene sets can then be used as input for traditional partial deconvolution techniques that require sets of tissue-specific genes. Figure 1d shows the application of the Digital Signal Algorithm (DSA)^3^ to these gene sets. This approach yields both the proportions and transcriptional profiles of the pure components within each mixture with a very high level of accuracy (Fig. 1d). This illustrates that leveraging the mutual linearity of cell-specific genes reveals the composition of cell mixtures in terms of both its components and their proportions without any a priori knowledge about either. It is important to note that this approach only reveals the cell types that vary within the cohort of the samples and does not discriminate between cellular subtypes that vary in the exact same way across all samples. However, this caveat is intrinsic to all complete deconvolution approaches.

### Row-normalization aligns mutual linearity to identity line

**Fig. 2:**
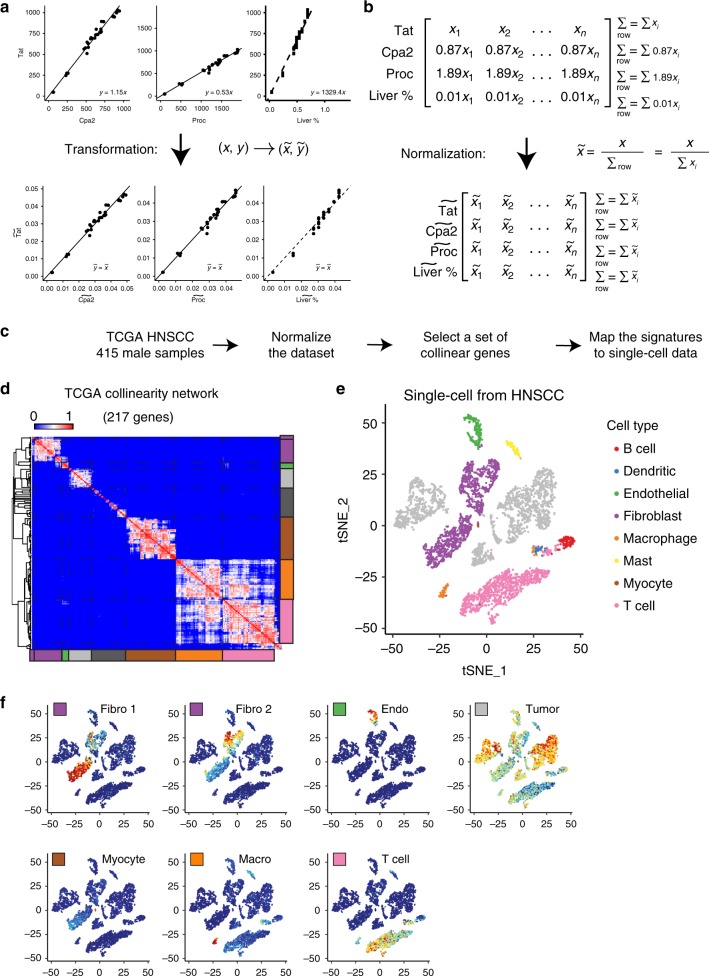
Gene collinearity of bulk HNSCC TCGA data reveals pure cell types consistent with scRNA-seq data. **a** Regression lines and coefficients before and after normalization. Regression line coefficient will change to unity in case of true signature genes. **b** In case of true signature genes normalized vectors will be equal. **c** Workflow for TCGA samples analysis and validation with the scRNA-seq data. **d** Left: TCGA collinearity network after filtration: seven clusters of highly collinear genes and dark-gray cluster that contains small 2–3 genes clusters. **e** Single-cell data for HNSCC reanalyzed (GSE103322). **f** Expression profiles of genes in seven clusters in the scRNAseq dataset, color represents averaged *Z*-score for genes in cluster from blue (min) to red (max)

Practically speaking, mutual linearity is assessed as the ability of the expression of two genes to obey a ${\mathbf{y}} = k \cdot {\mathbf{x}}$ fit, with the proportionality coefficient optimized for each pair of genes **y** and **x**. Naturally, the need to optimize the proportionality coefficient *k* for all possible gene pairs (i.e. $\sim 10,000 \times 10,000 = 10^8$ combinations) introduces considerable uncertainty to the process of searching for tissue/cell-specific genes. To eliminate this complication, we introduce a transformation such that all genes specific to one cell type become mutually linear with the coefficient *k* = 1 (Fig. 2a). For instance, consider the genes that are specific to liver tissue in GSE19830—*Tat*, *Proc*, etc. Since they are connected by the mutual linearity relationship $\left( {{\mathbf{y}} = k \cdot {\mathbf{x}}} \right)$, the expression values for *Proc* in each sample can be obtained by multiplying expression of *Tat* by an appropriate proportionality coefficient (e.g. by 1.89 in Fig. 2b). Therefore, the sum of all of the expression values in the row (i.e. across all samples) will differ by the same multiplication coefficient (Fig. 2b). Hence, if we normalize each expression value by the sum over the row, these multiplication coefficients will cancel out, yielding a *row-normalized* expression table where all the genes specific to one tissue are described by an identical vector (Fig. 2b). This transformation significantly simplifies the search for tissue specific genes, as it is sufficient to evaluate the accuracy of ${\tilde{\mathbf{y}}} = {\tilde{\mathbf{x}}}$ fit for all gene pairs.

Of note, if row-normalization is applied to a vector of cell type proportions **p**, it yields the normalized vector ${\tilde{\mathbf{p}}}$ that is also identical to row-normalized vectors of the genes ${\tilde{\mathbf{x}}}$ specific to this cell type (Fig. 2b). This correspondence reveals that the same mutual linearity relationship that exists between the expression of tissue-specific genes also extends to the cell type proportions (Fig. 2a, b).

### Mutual linearity reveals cellular populations in HNSCC tumor

We illustrate the power of the proposed approach by dissecting cellular heterogeneity within tumor samples (e.g. TCGA^16^). The work by Puram et al.^17^ dissected head and neck squamous cell carcinoma (HNSCC) tumors at single-cell resolution, explicitly describing transformed and non-transformed cell types within this tumor type, thus providing the ground truth for the cell type composition of HNSCC tumors. We applied our approach on the bulk whole tumor gene expression profiles of 415 samples from the HNSCC TCGA cohort and then used single-cell RNA-seq data to validate the deduced cell types within this tumor environment (Fig. 2c). The TCGA dataset was first trimmed to keep only 10,000 well-expressed genes and then row-normalized. For all pairs of row-normalized genes, we evaluated the extent of their linearity and kept 217 genes that have strong linear relationships (see Methods). Clustering these genes revealed seven major clusters that accumulated mutually linear genes (Fig. 2d). These clusters tentatively corresponded to the individual cell types that make up the tumors. To validate this result, we re-analyzed single-cell RNA-seq data from Puram et al. (GSE103322 (ref. ^17^). As Fig. 2e shows, 5902 cells separate into tumor cells, endothelial cells, fibroblast, myocyte, and immune cells. We then mapped the genes from each of the seven linear clusters obtained from the TCGA data onto the single-cell RNA-seq data. Indeed, as Fig. 2f shows, each of the linear clusters was enriched in an individual subpopulation, revealing myocytes, macrophages, two distinct fibroblast subtypes, endothelial and immune cells (mostly T cells), as well as genes specific to tumor subpopulations (Supplementary Data 2).

### Transformed gene expression space forms simplex

**Fig. 3:**
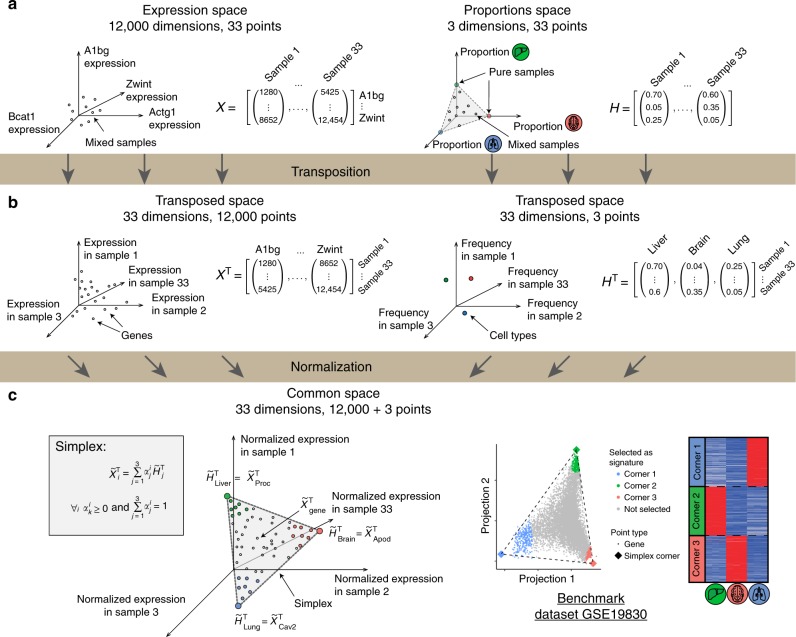
Derivation of the Transcriptional Simplex Lemma. **a** Samples are points in the high-dimensional gene expression space (left) or in the proportion space (right). **b** Transposed space contains genes as points where each gene is described by vector of its expression in 33 mixed samples (left); or pure cell type as points and each cell type is described by its proportions across 33 mixed samples (right). **c** Normalization aligns points from left and right parts of panel **b**: all expressed genes will lie within a simplex, corners of which are normalized cell type proportions (see proof in Supplementary Material). Bottom right: illustration of a simplex structure in GSE19830 dataset. One hundred genes closest to each corner were taken and expression profiles of these genes in pure samples are shown. Expression levels are from 0 (blue) to gene max (red)

Mutual linearity of cell-type specific genes suggests that the space of the mixed gene expression profiles might have a distinct underlying structure. Thus, we systematically investigated the topological properties of this gene expression space. A complete gene expression table is a collection of *N* vectors, where *N* is the number of profiled samples (e.g. 33 in the case of GSE19830), yielding a matrix **X** (e.g. 12,000 × 33 dimensions, see Fig. 3a, assuming ~12,000 well-expressed genes). Similarly, the composition of a mixed sample is described by a vector of the proportions of pure cell types, and the complete collection of mixed samples is described by ***N*** such vectors, yielding matrix **H** (3 × 33 dimensions in case of GSE19830, see Fig. 3a, right side). The convergence between the row-normalized expression of cell-type-specific genes and cell type proportions (see discussion around Fig. 2a, b) suggests that there might be a common space in which both vectors co-exist. Indeed, the rows of both matrices, **H** and **X**, have the same dimensionality—equal to the number of samples in the dataset, *N*. This means that the vectors that make up the transposed matrices **H**^T^ and **X**^T^ have the same dimensionality, and can be mapped as points within the common *N*-dimensional space. In total, matrix **H**^T^ will contribute as many points as there are pure cell types (3 in the case of GSE19830) and matrix **X**^T^ will contribute as many points as there are genes in the gene expression table (e.g. ~12,000) (Fig. 3b).

The convergence of row-normalized vectors of expression and cell proportion can be readily visualized in this *N*-dimensional space: when matrices **H** and **X** are row-normalized and then transposed (or first transposed and then column-normalized), the points described by vectors ${\tilde{\mathbf{H}}}_{{\mathrm{liver}}}^{\mathrm{T}}$, ${\tilde{\mathbf{H}}}_{{\mathbf{brain}}}^{\mathbf{T}}$, and ${\tilde{\mathbf{H}}}_{{\mathbf{lung}}}^{\mathbf{T}}$ will be identical to the points described by the vectors of tissue-specific genes from the matrix ${\tilde{\mathbf{X}}}^{\mathrm{T}}$ (e.g. ${\tilde{\mathbf{X}}}_{{\mathrm{proc}}}^{\mathrm{T}}$; Fig. 3c). This convergence is, in fact, a reflection of the very specific topological structure of the matrix ${\tilde{\mathbf{X}}}^{\mathrm{T}}$ in the *N*-dimensional space. Specifically, we find that all the points described by vectors in ${\tilde{\mathbf{X}}}^{\mathbf{T}}$ lie on a $(K - 1)$-dimensional simplex in the *N*-dimensional space, with *K* being the number of pure cell types and *N* being the number of samples in the dataset. For the GSE19830 dataset, given the row-normalized and transposed expression table (~12,000 × 33 dimensions), all of the ~12,000 points in the 33-dimensional space should lie within a triangle—a two-dimensional simplex enclosed by three vertices (Fig. 3c). In more accurate terms, one can formulate the following *Transcriptional Simplex Lemma*: the row-normalized gene expression vector for any gene *i* $\left( {{\tilde{\mathrm{X}}}_{ \ast ,{{i}}}^{\mathrm{T}}} \right)$ can be represented as a linear combination of the pure cell type row-normalized proportion vectors $\left( {{\tilde{\mathrm{H}}}_{ \ast ,{{j}}}^{\mathrm{T}}} \right)$ with non-negative coefficients $\alpha _j$ that sum to one (Fig. 3), i.e. they form a *K*−1 dimensional simplex in *N*-dimensional space. The rigorous proof of this statement is provided in the Supplementary Note 1 but, intuitively, each gene can be represented as a linear combination of cell proportions and appropriate normalization collapses all cell-type-specific genes and proportions into single points that become the corners of a simplex.

### Transcriptional simplex reveals signatures and proportions

The Transcriptional Simplex Lemma formulated above provides a direct and systematic approach to dissect the composition of a compendium of mixed samples: given expression table **X** for many mixed samples, one has to (a) row-normalize and transpose it yielding ${\tilde{\mathbf{X}}}^{\mathrm{T}}$, which can then (b) be analyzed to find the simplex hyperplane and its corners, where (c) the corners of the simplex define cell-type-specific signatures and cell proportions. Indeed, recent developments in the field of spectral unmixing^18,19^ introduced a number of geometrical approaches to find a simplex and its corners in multidimensional space (see simplex in Fig. 3c). We tested three main geometrical methods developed to date: MVSA^20^, SISAL^21^, and VCA^22^. We find that the SISAL algorithm is most robust, even for noisy data, and can identify the true simplex structure even in the absence of highly tissue-specific signature genes (Supplementary Fig. 1).

**Fig. 4:**
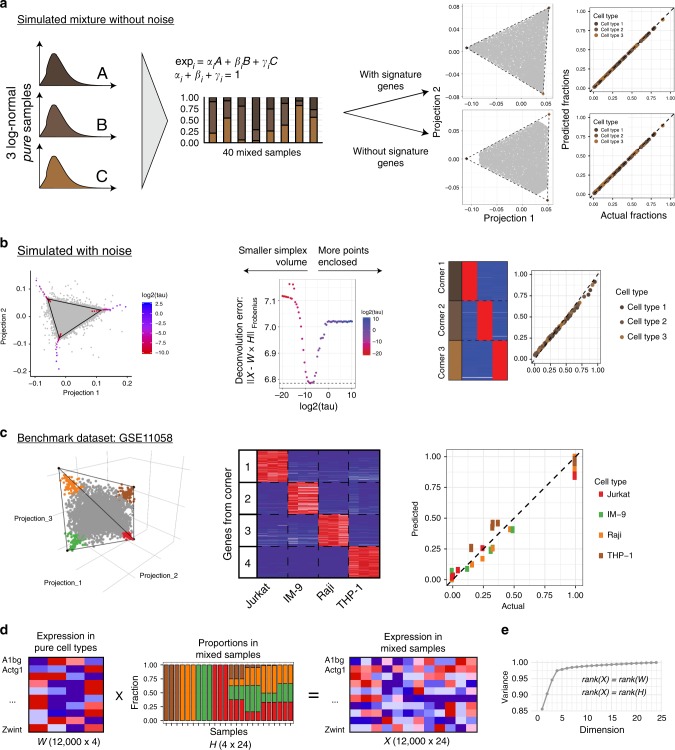
Geometric algorithms for simplex identification accurately solve the complete deconvolution problem in benchmark and simulations datasets. **a** Application of the algorithm to a simulated mixture. Left: dataset design—mixture of three pure samples with log-normally distributed gene expression. Right: Identification of simplex corners with and without signature genes: SISAL robustly identifies corners in both cases. **b** Dependence of deconvolution accuracy on tolerance level (tau) for simulated data with noise (left and middle). Deconvolution results for best tau (right): heatmap of corner genes expression in pure cell types and comparison of actual versus estimated cell fractions. **c** Application of algorithm to benchmark dataset GSE11058: identified simplex has a form of tetrahedron (4-simplex), heatmap shows cell-specific expression of corner genes and dot plot shows concordance between actual and estimated fractions of cell types. **d** Schematic for linear model for dataset GSE11058. **e** Variance explained by each SVD component for benchmark dataset GSE11058. Expression levels in heatmaps in panel **b** and **c** are from 0 (blue) to gene max (red)

To illustrate the geometric approach to simplex identification, we have computationally mixed three pure samples to obtain a panel of 40 different mixtures. Expression profiles of the pure cells types were obtained by independently simulating expression of ~12,000 genes in accord with log-normal distribution. As Fig. 4a illustrates, in such idealized mixtures, SISAL readily finds a two-dimensional subspace with genes enclosed into a triangular simplex. Genes selected from the corners of the simplex are selectively expressed pure cell types (Supplementary Fig. 2). The corners of the simplex are also the vectors of row-normalized proportions and thus yield a precise reconstruction of the pure cell type frequencies in the mixtures (Fig. 4a). Importantly, if all cell-type-specific genes are removed from the simulation dataset and the resulting simplex lacks points in its corners (Fig. 4a), the geometric approach to simplex identification still yields an accurate reconstruction of such mixtures, even when they lack explicit signature genes (Fig. 4b). This is particularly important in a biological context, where related cell types may lack robust signatures that uniquely discriminate them (e.g. monocytes and neutrophils, or erythrocytes and megakaryocytes).

### Noise-robust identification of the transcriptional simplex

In this section, we show that geometric simplex based deconvolution provides a natural way to account for noise in the data and parse out the linear signal coming from the mixing process. To that end, we first created simulated mixtures following the same scenario as in the previous section, but this time we added an independent white noise component to the expression of each gene (see Methods). As Fig. 4b shows, noise leads to blurred boundaries of the transcriptional simplex, which introduces uncertainty into the precise position and dimensionality of the simplex. In fact, SISAL provides a noise-dependent procedure for identification of simplex corners that is controlled by the single noise-tolerance parameter *tau*: larger *tau* values lead the algorithm to include as many points as possible inside the simplex, while smaller *tau* values minimize the volume of the simplex, discarding external points as noise (Fig. 4b, Supplementary Fig. 3). Therefore, depending on the choice of *tau* one can end up with a very different simplex. To choose the optimal *tau* value, we can compare the deviation between an experimental expression matrix (**x**) and a reconstructed matrix (**W** **×** **H**) obtained from the deconvolution process. Accuracy of reconstruction will be different for different *tau* (Fig. 4b, middle panel). The optimal value of noise tolerance *tau* can then be readily determined based on the accuracy of reconstruction. As Fig. 4b (right panel) shows, an optimal value of *tau* yields accurate cell-type-specific genes and correspondingly accurate cell-type proportions. Beyond simulated mixtures, application of this proposed approach to the benchmark dataset GSE11058 readily reveals a tetrahedral simplex structure in accord with the fact that this dataset is composed of four distinct cell types. Plotting expression of the corner genes in the pure samples reveals that they are highly cell-type-specific and yield accurate proportions (Fig. 4c).

### Singular value decomposition estimates number of cell types

One important aspect of all deconvolution methods is that they require knowledge of the number of pure cell types that make up the mixture. Fortunately, understanding the linear structure of the transcriptional space provides a direct way to infer the number of linearly independent components that contribute to variation in the dataset. In the idealized scenario, the matrix of the expression data **X** is the product of the matrices of pure cell type signatures **W** and corresponding proportions matrix **H** (Fig. 4d). Both **H** and **W** are matrices of rank *N* (number of pure cell types); accordingly, their product is a matrix of the same rank *N*. Therefore, if we can compute the effective rank of matrix **X** of mixed gene expression data, we can immediately infer the number of pure cell types in the mixture. In practice, there are two limitations: (1) gene expression matrix **X** is a non-square matrix, and traditional eigenvalue-based approaches are not applicable; (2) matrix **X** inevitably contains noise, and therefore the rank of *X* cannot always be defined precisely. These limitations can be circumvented to some extent by using Singular Value Decomposition (SVD) (Methods). For instance, Fig. 4e shows the cumulative variance explained by singular vectors obtained by SVD of the gene expression matrix of mixed samples from Fig. 4c, which immediately reveals that there are four major linearly independent components that define this gene expression matrix.

### Linear filtering improves deconvolution of noisy datasets

**Fig. 5:**
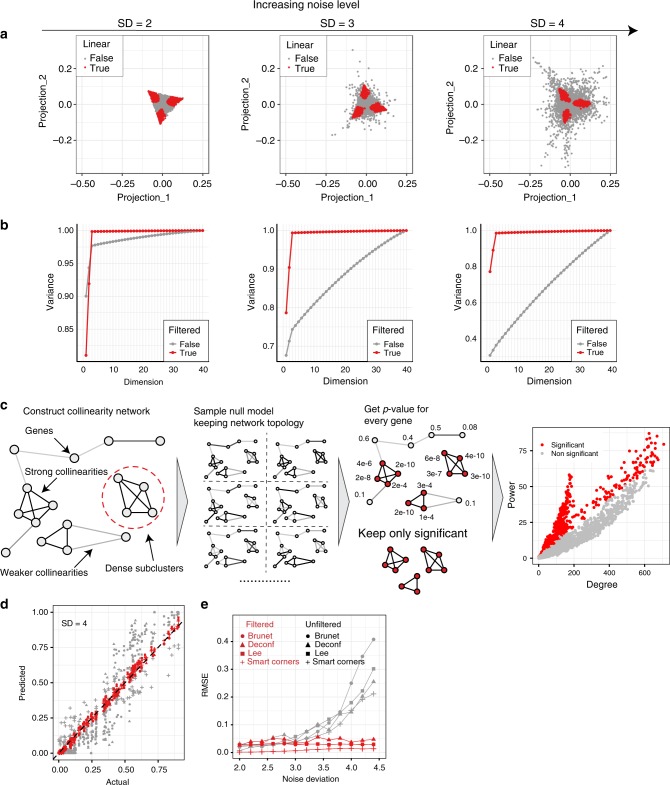
Mutual linearity filtering improves deconvolution methods and allows cell type number identification in noisy cases. For panels **a** and **b** noise level is increasing from left to right. **a** Two-dimensional projection of simulation with noise. Points in red are selected by filtering procedure. **b** Variance explained by each SVD component for both unfiltered (gray) and filtered (red) datasets. **c** Schematic for filtering approach. Collinearity network is built by calculating all the pairwise linearity coefficients and spearman correlations. When the network is built, weights of the edges are randomly shuffled to test each gene to have more total weight than at random. We select only those genes with a significance level < 0.01. On the right is illustration of which genes are selected, where power denotes the sum of the weights of the edges. **d** For very noisy dataset linearity filtering improves performance of all complete deconvolution methods. **e** For different noise levels RMSE of predicted and actual proportions is calculated

Noise that arises in real datasets due to imperfect mixing and/or biological perturbations can often be prohibitively large to readily reveal the linear subspace of gene expression data. Figure 5a, b illustrates this point using simulated mixtures of three components with various levels of noise (see gray dots/graphs). Mathematically, non-zero singular vectors beyond the number of cell types arise because SVD attempts to fit the non-linear variation with linear components which are not relevant for the complete deconvolution procedure. Thus, we next focused on devising an unbiased approach to identify a subset of mutually linear genes for any given dataset (Fig. 5c).

We first constructed a mutual linearity network by connecting all pairs of genes with edges weighted by both their mutual linearity coefficient and their spearman correlation (see Methods). Then, we performed null model simulations by maintaining the network topology while permuting the weights of the edges. These simulations yield a *p*-value for each gene, defined as a the probability to observe combined mutual linearity of all edges associated with the gene. The genes above the statistical significance cut-off (0.01; Fig. 5c, right panel) form the set of mutually linear genes. SVD and deconvolution procedures are then applied to this set of genes. Red dots in Fig. 5a illustrate the positions of such genes and show that at all noise levels, the filtering procedure robustly identifies corner-specific genes and filters out irrelevant noisy genes. Red bars in Fig. 5b show that this filtering procedure effectively removes the non-linear components of noise and provides an accurate estimation of the number of cell types, even when the use of a non-filtered dataset results in a completely inconclusive SVD decomposition.

This filtering procedure provides an important pre-processing step that can be highly beneficial for all complete deconvolution approaches, not just the ones that are based on simplex identification. Indeed, when we applied a mutual linearity-based filtering step prior to the *brunet*, *deconf*, and *lee* deconvolution approaches, it significantly improved the ability of the algorithms to reconstruct the data in all cases, even with high levels of noise (Fig. 5d, e). Thus, we conclude that revealing the mutual linearity of tissue/cell-specific genes has a significant impact on deconvolution approaches and advances our ability to perform complete deconvolution on noisy biological datasets.

### Complete deconvolution pipeline

**Fig. 6:**
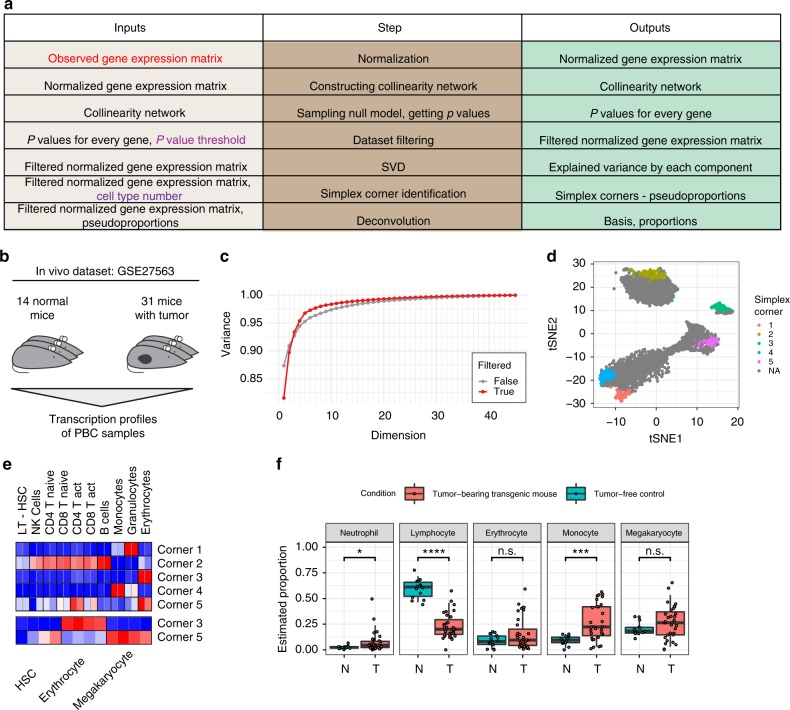
Complete deconvolution pipeline is able to dissect realistic datasets. **a** Pipeline steps required to perform complete deconvolution. **b** Schematic of GSE27563 dataset. **c** First five components of SVD of the filtered dataset explain more that 97% of the variance. Closest 100 genes to each simplex corner for panels **d** and **e**. **d** TSNE dimensionality reduction can be used to highlight the structure of high-dimensional filtered dataset. **e** We used datasets GSE27787 for mouse hematopoietic cells, and GSE49664 for erythrocyte-related population to identify cell types in the mixture. Heatmaps show averaged *z*-score of identified gene sets in population presented in the datasets. Values are from gene set minimum (blue) to gene set maximum (red). **f** Box and dot plot for deconvolution results, showing changes between normal and tumor-bearing mice. For each boxplot bottom whisker, bottom of the box, middle line, top of the box, and top whisker are 5%, 25%, 50% (median), 75%, and 95% quantiles respectively. Group comparisons were determined using a two-sided Mann–Whitney *U* test (n.s. *p* value >0.05, *p value <0.05, ***p value <0.001, ****p value <0.0001)

Altogether, the mutual linearity concepts described in the previous sections amount to the following pipeline for complete deconvolution (Fig. 6a). First, gene expression samples are row-normalized. Next occurs filtering the dataset based on the mutual linearity of the genes. Then, SVD-based analysis defines the putative number of cell types that vary in the mixture. At this point, a simplex of known dimensionality is constructed using procedure defined in Fig. 4b, and the corners of the simplex provide information about cell type proportions and cell-type-specific genes within the mixture. Application of this pipeline to benchmark datasets GSE19830 and GSE11058 yields very accurate deconvolutions (Supplementary Figs. 4, 5).

To further illustrate the complete deconvolution pipeline, we analyzed dataset GSE27563 (ref. ^23^), where mouse blood was profiled from animals with and without tumors (total 45 mice; Fig. 6b). After filtering (Supplementary Fig. 6), the gene expression matrix consists of 2674 significantly mutually linear genes, and can be described as a mixture of five cell types (Fig. 6c). Consistently, when filtered genes are mapped onto a tSNE projection, putative cell-type-specific genes fall in five distinct clusters (Fig. 6d). These signatures were then compared against a dataset of pure murine blood cell types—GSE6506 (ref. ^24^). This analysis revealed lymphocytes, monocytes, and granulocytes as well as two different subtypes with enriched erythrocytic signatures (Fig. 6e). We have further used GSE49664 (ref. ^25^), where murine erythroid cell subpopulations were profiled, and we found that corner two genes corresponded to megakaryocytes, while corner three genes corresponded to classical erythrocytes. Thus, we identified five major blood cell types in murine blood—erythrocytes, megakaryocytes, lymphocytes, monocytes, and granulocytes (neutrophils). Consistent with biological expectations, blood of tumor-bearing animals contained a significantly higher proportion of monocytes and a lower proportion of lymphocytes (Fig. 6f).

Next, we re-analyzed the HNSCC TCGA dataset used in Fig. 2 to illustrate the relevance of mutual linearity to real large-scale datasets. We find that after filtering there remain 680 mutually linear genes, which can be described by the four main cell types. When signatures of these four cell types are mapped onto single-cell RNA-seq data, they identify as cancer cells, immune cells, myoblasts, and fibroblasts (Supplementary Fig. 7e). The frequencies of immune cells obtained from our approach match (Supplementary Fig. 7f) the ones computed by TIMER^13^, the state of the art tool for computational deconvolution of immune infiltration into tumor tissues, which was trained on immune signatures. This confirms the ability of mutual linearity-based complete deconvolution to accurately dissect both cellular composition and cellular frequencies in large-scale datasets and those with significant noise level, while the existing complete deconvolution approaches fail to successfully analyze datasets of this level of complexity in the absence of the mutual linearity filtering step (Supplementary Fig. 8).

Finally, we applied the same pipeline to human blood samples (PBMCs) collected from 13 healthy volunteers at 0, 3, and 7 days post-vaccination (Supplementary Fig. 9). When patients were given the MCV4 vaccine formulation, a considerable spike in plasma cell abundance was observed^26^. We analyzed PBMC gene expression for these patients to evaluate the predictive power of our approach. We found that the collection of 39 samples could be described as a mixture of four cell types and used simplex-based deconvolution of the gene expression matrix to identify cell-type-specific signatures. Comparing the signatures with a differentiation map of hematopoiesis^27^ (DMAP) and dataset GSE45535(ref. ^28^), we identified the deconvolved cell types as monocytes, T cells, erythrocytes, and plasma cells (Supplementary Fig. 9). Consistent with general blood composition, lymphocytes and monocytes were the predominant cell populations among PBMCs and the proportions of the plasma cells systematically increased on day 7 after vaccination, in accord with the FACS measurements reported in Li et al.^26^.

### Systematic error due to difference in cellular RNA content

**Fig. 7:**
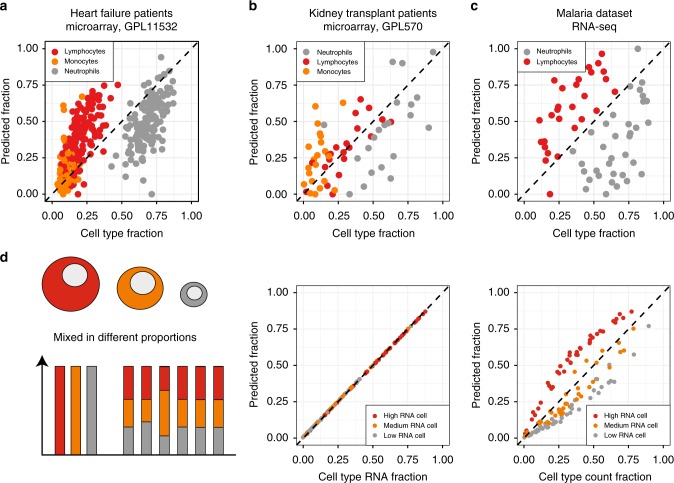
Complete deconvolution pipeline reveals bias when fractions are compared to blood counts. The results of the pipeline for dataset GSE20300 (**a**), GSE77434 (**b**), and E-MTAB-6413 (**c**). **d** Simulation dataset where cell types had different RNA content. In the middle, predicted proportions are compared against fraction of cell types RNA in the sample. On the right, predicted proportions are compares against actual cell type counts

Next, we benchmarked the mutual linearity-based deconvolution approach against experimental datasets where cellular proportions were directly defined by FACS measurements. We focused on three datasets profiling whole blood: GSE20300, GSE77343, and E-MTAB-6413 (Fig. 7a–c). These datasets contained the data on 24, 142, and 39 donors, and were profiled using two different microarray platforms or RNA-sequencing (HGU133V2, Human Gene ST Array, and RNA-seq respectively). In all cases (see Supplementary Figs. 10–12) linear variance was reasonably well explained by three or four cellular components, which were identified as neutrophils, lymphocytes, monocytes, and erythrocytes based on comparison with the pure cell type compendiums DMAP and GSE45535. The partial deconvolution algorithm CIBERSORT^5^ has been optimized for the HGU133 platform, and for the GSE20300 dataset cellular frequencies obtained by our approach compared very well with CIBERSORT (Supplementary Fig. 10f–h). However, even though cell signatures correctly identified cell types, we observed (Fig. 7a–c) that a fraction of lymphocytes was always systematically overestimated in our deconvolution approach, while a fraction of neutrophils was always underestimated, independent of the platform or clinical context. We hypothesized that this could be due to the difference in cell sizes and associated RNA-per-cell content of these cell types, as it is well known that neutrophils generally carry much lower RNA quantity than lymphocytes. Indeed, simulation of a mixing model with variable RNA per-cell content shows that a difference in cell size or cellular RNA content can lead to such systematic differences when comparing to true cell type proportions (Fig. 7d).

**Fig. 8:**
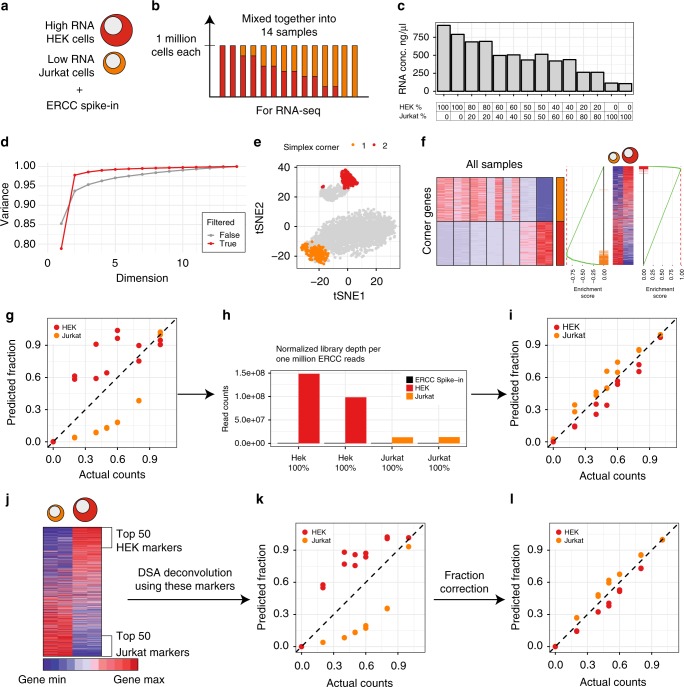
Systematic error is caused by difference in cell size/cellular RNA content. Experiment schematic: **a** cell types that were chosen for the experiment, **b** proportions of the cell type for the experiment. **c** RNA content for each sequenced sample. **d** Explained variance by SVD for filtered and unfiltered datasets. Closest 100 genes to each simplex corner were selected to identify cell types. **e** TSNE plot for the filtered dataset: two clusters can be visually identified. Colored genes are those from the corners. **f** Gene expression profiles of selected genes in mixed samples and GSEA comparing these genes to differential expression between pure samples of HEK and Jurkat cells. **g** Proportions predicted by the pipeline. **h** Bar plot showing relative RNA content to ERCC-spike-in. **i** Corrected proportions of the pipeline. **j** Usual pipeline for complete deconvolution: we detect the markers using differential expression and use these markers as input of DSA algorithm. **k** Proportions predicted by DSA using differential expression markers. **l** Corrected proportions of **k**

To validate the effect of cell size or RNA content, we prepared mixtures of two cell types of distinctly different sizes and cellular RNA content: HEK and Jurkat cells (Fig. 8a, b), always ensuring that each mixture contained total of one million cells. Consistent with expectations, the total RNA yield from these samples correlated with the fraction of HEK cells (Fig. 8c). We then performed RNA-sequencing of these samples (including ERCC spike-in controls to be able to control for the absolute RNA-concentration), and analyzed the data using our proposed complete deconvolution approach (see Fig. 6a). Indeed, SVD analysis (Fig. 8d) applied after linear filtering (Supplementary Fig. 13) revealed that the mixture was composed of two cell types, and mutually linear genes clustered into two major clusters associated with the genes derived from the corners of the two-dimensional simplex (Fig. 8e). The inspection of these corner genes revealed that they were distinctly cell type specific (Fig. 8f). However, reconstructed cell proportions did not match the actual cellular frequencies used in the experimental design (Fig. 8g). This was consistent with the idea that cell size difference will introduce a systematic error. Such an error can be compensated provided that the relative RNA per cell content is known for the cells in the mixture. In this case, the information can be derived by normalizing the data to spike-in ERCC controls, and by comparing library depth in pure HEK and pure Jurkat cells (Fig. 8h). This comparison shows that mRNA content in HEK cells is approximately six times higher than in Jurkat cells. Introducing this coefficient into the deconvolution data (see Methods) leads to an excellent agreement between the predicted cell proportions and the actual cell counts (Fig. 8i). Our approach has significantly outperformed NMF-based approaches on this real dataset (Supplementary Fig. 13a), and filtering of the mutually linear genes in accord with mutual linearity improved the performance of NMF methods, consistent with the simulation results (Supplementary Fig. 13b). Importantly, the bias was evident also when using partial deconvolution approaches (Fig. 8j) with known cell type markers (Fig. 8k), which can be corrected by using the spike-in derived coefficient (Fig. 8l).

## Discussion

In summary, we describe a noise-robust approach to solving the complete deconvolution problem that reveals the composition of mixed samples based on their bulk gene expression profiles and without any a priori knowledge about the pure components. Provided that the input dataset is large enough to faithfully capture the linear component of the variability across multiple samples, our approach works robustly with and without noise and performs well on both benchmark and complex tissue datasets. Natural restrictions to this approach include very small cohort samples, or noise so large that it masks the linear component of the variability. This can happen, for instance, if a particular cell type has a very low abundance (compared to the level of technical noise). Likewise, for subpopulations that are transcriptionally very close to each other, variability in the proportions of these subpopulations must be larger than the degree of their transcriptional similarity. Finally, in the case when two different cell types co-vary with each other across all samples, our approach will not be able to discriminate individual cell types, but rather will view them as one supertype.

Overall, the presence of the topological structures that we reveal has been alluded to previously by Uri Alon’s group^29^, where they show that, broadly speaking, biological tasks can be considered as polytopes in a multidimensional space. The presence of the simplex topology in mixed gene expression was also noted by Wang et al.^9^ based on an analogy with hyperspectral image decomposition. In this context, our work provides an explicit description of this type of transformation and its underlying biological meaning (mutual linearity of tissue-specific genes) coupled with a geometric approach for simplex identification that allows robust identification of tissue-specific genes or their proxies.

One advantage of geometrical methods for simplex identification is that both tissue-specific genes and genes shared across samples significantly contribute to simplex identification, as they allow to establish the hyperplane where the simplex is located and then find simplex boundaries. A potentially more important advantage of geometrical methods is that they are able to identify a proper simplex even in situations when mixed cell types do not have explicit signature-genes. This strategy, while conceptually simple, is dramatically different from the one used by Wang et al.^9^, who did not search for vertices of the simplex but rather considered all clusters of the co-expressed genes by using an affinity propagation algorithm and then tested all possible combinations of these clusters to find the optimal combination that reconstructs a dataset with the smallest error margin (Supplementary Fig. 14).

As we point out in Fig. 5, the simplex-based deconvolution and published NMF-based complete deconvolution approaches provide equally accurate solutions for both benchmark datasets and idealized simulation datasets but behave differently in the situation of realistic levels of noise. Ability to filter out genes that are non-cell-specific allows one to efficiently work with datasets of arbitrary scale and realistic levels of noise, as we highlight using the example of the TCGA datasets (see Supplementary Fig. 8). The ability to dissect large biological datasets is particularly important as many marker-based deconvolution approaches are optimized to perform with specific profiling platforms (e.g. CIBERSORT^5^), or in particular biological settings, such as tumor-infiltrating immune cells (e.g., TIMER^13^).

## Methods

### Downloaded microarray datasets

Normalized microarray data were downloaded from the Gene Expression Omnibus (GSE11058—controlled mixtures of human immune cell lines, GSE19830—controlled mixtures of rat brain, liver and lung, GSE19380—controlled mixtures of brain cell subsets, GSE27563—expression data from murine PBCs from mice with advanced mammary tumors and their tumor-free counterparts, GSE52245—time course of young adults vaccinated with meningococcal mcv4 and mpsv4, GSE20300—whole blood gene expression analysis of stable and acute rejection pediatric kidney transplant patients), GSE77343—whole blood gene expression in chronic heart failures.

Cell-specific transcriptional profiles that were used for enrichment were obtained from GSE27787 for mouse hematopoietic cells, GSE49664 for primary megakaryocytes and erythroblasts from murine fetal liver hematopoietic stem/progenitor cells, GSE45535 for human blood subsets including plasma cells, and DMAP for normal human blood subsets (especially for erythrocyte contamination).

### Microarray datasets pre-processing

As microarray data contain a lot of noise, some preprocessing steps were applied before deconvolution analysis. We use the following steps to extract the signal and avoid unwanted noise:

Collapse probes by gene symbol: remove probes mapping to several genes, if several probes associated to the same gene, use a probe with a maximum average expression as representative.

Use log-transformed values to calculate an average expression for each gene.

Choose top 12000 highest expressed (on average) genes.

Remove artificial sources of linearity: genes from sex chromosomes (if samples are not sex-matched) and ribosomal component genes (RPL/RPS).

Remove sample outliers if necessary.

Perform quantile normalization if the dataset was not normalized.

For all downstream computations, we use linear-transformed (non-log) expression values.

### TCGA data preprocessing

The gene per sample expression matrix of HNSCC from TCGA was downloaded from https://software.broadinstitute.org/morpheus/. Only non-protein coding genes and RPL/RPS genes were removed from the dataset (15,807 gene symbols left). The dataset was then linear-transformed, and samples were normalized to have the same sum of expression levels. Only the top 10,000 highly expressed genes by average expression were kept for the analysis. Only male samples were kept for the analysis.

### Cell cultures

HEK-293T were obtained from ATCC (ATCC CRL-321666) and cultured in DMEM supplemented with 10% fetal bovine serum (FBS), 2 mM l-glutamine, and 100 U ml^−1^ penicillin–streptomycin. Jurkat cells were provided by laboratory of Prof. Robert D. Schreiber and cultured in RPMI supplemented with 10% FBS, 2mM l-glutamine, and 100 U ml^−1^ penicillin–streptomycin. Both cell lines were passaged regularly twice per week. For experiment cells were harvested 2 days after last passage, pelleted, and resuspended in PBS containing 0.2% bovine serum albumin at concentration 10^6^ ml^−1^. Mixtures of given proportions were then prepared by mixing HEK-293T and Jurkat cell suspensions into final volume of 1 ml. Cell mixtures were then pelleted and further processed.

### RNA sequencing

mRNA was extracted from cell lysates by means of oligo-dT beads (Invitrogen). For cDNA synthesis, we used custom oligo-dT primer with a barcoded adaptor-linker sequence (CCTACACGACGCTCTTCCGATCT-XXXXXXXX-T15). After first-strand synthesis, samples were pooled together based on Actb qPCR values and RNA–DNA hybrid was degraded with consecutive acid–alkali treatment. Then, a second sequencing linker (AGATCGGAAGAGCACACGTCTG) was ligated with T4 ligase (NEB) followed by SPRI clean-up. The mixture then was PCR enriched 12 cycles and SPRI purified to yield final strand-specific RNA-seq libraries. Data were sequenced on HiSeq 2500 by 40bpX11bp pair-end sequencing. Second mate was used for sample demultiplexing.

### RNA-seq data acquisition and processing

Demultiplexed single-end fastq files were aligned to the mixture reference GRCh38 and ERCC spike-in sequences by top-level assembly with STAR (version 2.6.1b). Gene counts were produced RSEM (version v1.3.1).

We used Deseq2 R/Bioconductor package to obtain differential expression between pure samples of HEK and Jurkat cell lines. Differential expression was obtained by Deseq2 guidelines; all *p* values were corrected for testing multiple genes (Bonferroni correction). The top 100 genes (upregulated in a pure cells) were selected as cell type markers for DSA deconvolution.

### Simulation dataset

We simulated a $12,000\;{\mathrm{genes}}\; \times \;40\;{\mathrm{samples}}$ matrix of observed gene expression **X** of mixed samples by simulating two matrices: a 12,000 × 3 matrix **W** (gene signatures) and a 3 × 40 matrix **H** (proportions). We simulated **W** using log-normal distribution with a mean of 6 and standard deviation of 1.5 for each sample:

1 $$\begin{array}{*{20}{c}} {\forall i \in \left[ {1 \ldots 3} \right]{\mathbf{W}}_{ \ast,i}\sim 2^{N\left( {6,1.5} \right)}} \end{array}$$

and since proportions sum-to-one constraint is usually assumed in complete deconvolution problem, we can sample matrix **H** uniformly from the unit simplex using the approach described in^30^:

2 $$\begin{array}{*{20}{c}} {\forall j \in \left[ {1 \ldots 40} \right]{\mathbf{H}}_{ \ast ,i}\sim U\left[ {\Delta ^3} \right]} \end{array}.$$

Matrix **X** was simulated as multiplication of these two matrices plus log-normal Gaussian noise with zero mean:

3 $$\begin{array}{*{20}{c}} {{\mathbf{X}}_{{\mathrm{{SD}}} = k} = {\mathbf{W}} \times {\mathbf{H}} + 2^{N\left( {0,k} \right)},k \in \left[ {0..7} \right]} \end{array},$$

where SD and *k* are standard deviation and noise level. A model with SD = 0 was used in this paper as simulation data without noise. A model with SD = 4 was used as simulation data with noise, as the noisiest model that had distinguishable signal by SVD.

Simulation data without signature genes was obtained by removing tissue-specific genes—such *i* that$\sqrt {\mathop {\sum }\limits_{j = 1}^3 \left( {\widetilde {w_{i,j}}} \right)^2} \ge 0.85$, where $\widetilde {w_{i,j}} = \frac{{w_{i,j}}}{{\mathop {\sum }\nolimits_{j = 1}^3 w_{i,j}}}$.

For Fig. 5 we used samples from the Liver–Brain–Lung dataset (GSE19830) as pure samples instead of sampling log-normal matrix **W**. Proportions and noise were simulated the same way as above.

For simulation with different RNA content we simulated matrix **W** as described above, when we divided second column of **W** by two and third column by three. This gave us three cell types with different RNA content. We simulated **H** to meet sum-to-one constraint. We simulated **X** by multiplication of **W** and **H** and further normalization of columns of **X** to have equal column sum.

### Row normalization

Row normalization of dataset **X** is defined as a matrix ${\tilde{\mathbf{X}}}$ every row of which is a row of matrix **X** normalized by its sum, i.e.

4 $$\begin{array}{*{20}{c}} {\forall i \in \left[ {1,N} \right],j \in \left[ {1,M} \right]\;\tilde x_{i,j} = \frac{{x_{i,j}}}{{\mathop {\sum }\nolimits_{k = 1}^M x_{i,k}}}} \end{array},$$

where *N* is the number of genes and *M* is the number of samples.

### Collinearity networks

To measure linearity between to genes **x** and **y** we first normalize expression levels of these genes (${\tilde{\mathbf{x}}}$ and ${\tilde{\mathbf{y}}}$) and then we evaluate how well the line of ${\tilde{\mathbf{x}}} = {\tilde{\mathbf{y}}}$ fits the normalized expression values by calculating the average of the two coefficients of determination *R*^2^ for two models ${\tilde{\mathbf{x}}} = {\tilde{\mathbf{y}}}$ (${\tilde{\mathbf{x}}}$ is dependent and ${\tilde{\mathbf{y}}}$ is variable) and ${\tilde{\mathbf{y}}} = {\tilde{\mathbf{x}}}$ (${\tilde{\mathbf{y}}}$ is dependent and ${\tilde{\mathbf{x}}}$ is variable). Let us denote this linearity coefficient as $R_{{\mathrm{{sym}}}}^2\left( {{\mathbf{x}},{\mathbf{y}}} \right)$. Then we calculate spearman correlation between each pair of genes ${\mathrm{\rho }}\left( {{\mathbf{x}},{\mathbf{y}}} \right)$.

To build an undirected weighted linearity network, we use genes as nodes of the network. We put the edge between two genes *x* and *y* if both $R_{{\mathrm{{sym}}}}^2\left( {{\mathbf{x}},{\mathbf{y}}} \right) > 0$ and ${\mathrm{\rho }}\left( {{\mathbf{x}},{\mathbf{y}}} \right) > 0$. We set the weight of such edge to be

5 $$\begin{array}{*{20}{c}} {{\mathbf{W}}_{{\mathbf{x}},{\mathbf{y}}} = R_{{\mathrm{{sym}}}}^2\left( {{\mathbf{x}},{\mathbf{y}}} \right)\rho \left( {{\mathbf{x}},{\mathbf{y}}} \right)}. \end{array}$$

### Significance test

For any gene *x* in the network, let us denote set of outgoing edges as $E\left( x \right)$. Let us also denote sum of weights of $E\left( x \right)$ as power of *x*: $P\left( x \right) = {\sum}_{j \in E\left( x \right)} {\mathbf{W}}_{x,j}$.

We would like to find genes that have a power greater than at random taking into account the topology of the network. We test this null hypothesis for each gene by sampling weights of the network.

We first calculate powers in the actual network $P_{{\mathrm{{actual}}}}\left( x \right)$. Let *K* be the number of sampling iterations. Let ${\mathrm{{Success}}}\left( x \right)$ denote the number of successful samplings for gene x, when $P_{{\mathrm{{sampled}}}}\left( x \right) \ge P_{{\mathrm{{actual}}}}\left( x \right)$, vector ${\mathrm{Success}}\left( x \right)$ is initialized with zeroes. Each iteration we will randomly shuffle weights of the edges of the network while keeping the network topology and then calculate the sampled power of each gene: if the sampled power of the gene *x* is greater or equal to the actual power of gene *x*, we will increment ${\mathrm{Success}}\left( x \right)$ by one.

We can then calculate *p*-value for each gene

6 $$\begin{array}{*{20}{c}} {p\left( x \right) = \frac{{{\mathrm{{Success}}}\left( x \right) + 1}}{{K + 1}}.} \end{array}$$

This procedure allows robust identification of genes with collinear expression profiles and provides *p* values quickly. However, if one wants to adjust these *p* values for multiple comparison using Bonferroni correction, one must increase the number of sampling iterations: if *a* is the desired significance level and *N* is the number of genes in the network then $K > \frac{N}{{\mathrm{\alpha }}}$ is required to obtain the desired confident *p* values.

### TCGA dataset initial processing

Another way to build a linearity network is to calculate linearity coefficients between all pairs of genes and replace all negative values with zeroes. We then filter the matrix of all pairwise linearities by keeping only the genes that meet the requirements below:

Gene has at least *k*_1_ gene with linearity values of greater or equal to $\mathrm {threshold}_{1}$

Gene has at least *k*_2_ genes with linearity values of greater or equal to ${\mathrm{{threshold}}_{2}}$

The rationale behind these requirements is quite straightforward: we would like to get rid of genes that are not linear to any other genes and want to guarantee a finding of clusters of meaningful size. Usually, we set ${\mathrm{{threshold}}_{1}}$ to be greater than ${\mathrm{{threshold}}_{2}}$, and selection of these thresholds may vary from dataset to dataset.

We then hierarchically cluster the filtered matrix using 1-Pearson correlation as a distance and average linkage and this leads to the identification of linear subnetworks.

For the TCGA dataset from Fig. 2 we used $k_1 = 1,\,{\mathrm{{threshold}}_{1}} = 0.75$ and $k_2 = 10,\, {\mathrm{{threshold}}_{2}} = 0.25$. After filtration, only 217 genes were left, and hierarchical clustering identified seven modules with cell-type specific clusters and small modules that we were not able to assign to any specific cell type.

### Reconstruction accuracy

The complete deconvolution problem is a factorization problem, given **X** we try to find such factors **W** and **H** that will describe cell type expression signatures and cell type proportions. We estimate the accuracy of reconstruction (deconvolution accuracy) as the Frobenius norm of input and estimated multiplication:

7 $$\begin{array}{*{20}{c}} {\left( {{\mathrm{{input}}}} \right) {\mathbf{X}} \approx {\mathbf{W}} \times {\mathbf{H}}\left( {{\mathrm{{output}}}} \right)} \end{array}$$

8 $$\begin{array}{*{20}{c}} {{\mathrm{{accuracy}}} = {\mathbf{X}} - {\mathbf{W}} \times {\mathbf{H}}_{{\mathrm{{Frobenius}}}}}. \end{array}$$

### Algorithm

The algorithm takes as input matrix *X* of observed gene expression in mixed samples. The algorithm consists of several key steps which will be described below in detail:

Row normalization

Constructing collinearity network (described above)

Sampling null model and getting *p* values (described above)

Dataset filtering by *p* value

SVD and cell type number estimation

Simplex corner identification

Deconvolution

### Normalization

Normalization is one of the key features of the algorithm. In the proof of Transcriptional Simplex Lemma (Supplementary Material) we show that sum-to-one normalization in linear space (where each gene expression level is divided by its sum) puts all the genes in a simplex, the corners of which will are normalized cell type proportions.

### Cell-type number estimation

Cell-type number can be addressed with different approaches. First, one might a priori know or assume the number of major cell types in the mixture and use this number as the dimensionality of linear subspace. If this number is not known a priori, SVD can be used to estimate the effective rank of **X**: let us consider an observed gene expression *N* × *M* (*N* genes and *M* samples, *M* < *N*) matrix **X**, whose singular value decomposition is given by **X** = **UDV**, where **U** and **V** are *N* × *N* and *M* × *M* unitary matrices and **D** is a diagonal matrix containing singular values $\sigma _1 \ge \sigma _2 \ge \ldots . \ge \sigma _M$. The natural way to look at singular values is explained variance

9 $$\begin{array}{*{20}{c}} {\alpha _i = \frac{{\sigma _i^2}}{{\mathop {\sum }\nolimits_{j = 1}^M \sigma _j^2}}.} \end{array}$$

### Projection to a linear subspace

Projection to linear subspace is well-described by Nascimento et al.^22^. In brief, to project the dataset to a smaller linear subspace we first normalize it and transpose it, so genes are column vectors. We then calculate SVD for the zero-centered dataset. Once a number of cell types *k* is selected, the non-centered dataset is projected to the space generated by *k* –1 left-singular vectors:

10 $$\begin{array}{*{20}{c}} {{\tilde{\mathbf{X}}}_{{\mathrm{zero}} \mbox{-} {\mathrm{centered}}}^{\mathrm{T}} = {\mathbf{UDV}}^{\mathrm{T}},{\mathbf{U}}_{k - 1} = {\mathrm{first}}\;k - 1\;{\mathrm{singular}}\;{\mathrm{vectors}}\;{\mathrm{of}}\;{\bf{U}}} \end{array},$$

11 $$\begin{array}{*{20}{c}} {{\tilde{\mathbf{X}}}_{{\mathrm{projected}}}^{\mathrm{T}} = {\mathbf{U}}_{k - 1} \times {\tilde{\mathbf{X}}}^{\mathrm{T}}} \end{array}.$$

### Corner identification

We tested three different algorithms for their ability to identify simplex corners (Supplementary Fig. 1) and found that SISAL is robust to noisy data and has a parameter *tau* which allows to control for noise tolerance. We iterate through different *tau*

12 $$\begin{array}{*{20}{c}} {\forall i \in N\;{\mathrm{{and}}}\;i \in \left[ { - 20,0} \right],{{\mathrm{{tau}}}}_i = 2^i} \end{array}$$

and for each tau we select the corners and then use these corners to deconvolve the dataset and calculate the reconstruction error. We choose tau with the smallest reconstruction error.

We also implemented an approach called Smart Corners which allows to choose different tau for each of the cell types by calculating reconstruction errors for possible combinations of different tau for each of the corners and then choosing combination with the smallest reconstruction error.

### Signature gene selection

When the corner is identified, the Euclidean distance between every gene and every corner is calculated in the projected space. For each corner we can select *G* closest genes to each corner. These genes will help us to identify the cell type.

### Deconvolution

We perform deconvolution using simplex corners as putative signatures in a DSA-like manner. Let *k* × *m* matrix **H**_p_ be simplex corners (i.e. row-normalized **H**, actual cell type proportions), where *k* is the number of cell types and *m* is the number of samples. Then we first find such coefficients ${\mathrm{\alpha }}_1 \ldots {\mathrm{\alpha }}_k$ that would fit best the equation

13 $$\begin{array}{*{20}{c}} {\left( {\begin{array}{*{20}{c}} {{\mathrm{\alpha }}_1} \\ \vdots \\ {{\mathrm{\alpha }}_k} \end{array}} \right) \times {\mathbf{H}}_{\mathrm{p}} = \left( {111 \ldots 111} \right).} \end{array}$$

Then matrix **H** is calculated as $${\mathbf{H}} = \left( {\begin{array}{*{20}{c}} {{\mathrm{\alpha }}_1h_{1,1}^{\mathrm{p}}} & \cdots & {{\mathrm{\alpha }}_1h_{1,m}^{\mathrm{p}}} \\ \vdots & \ddots & \vdots \\ {{\mathrm{\alpha }}_kh_{k,1}^{\mathrm{p}}} & \cdots & {{\mathrm{\alpha }}_kh_{k,m}^{\mathrm{p}}} \end{array}} \right)$$, where $h_{i,j}^{\mathrm{p}}$ are elements of **H**_p_ Matrix **W** is then calculated using fast combinatorial non-negative least squares.

### Fraction correction

Let **W**^cell^ be an *N* × *K* matrix of true gene expression profiles for one cell for each cell type, i.e. each column of **W**^cell^ represents an average cell of a given cell type and different columns of **W**^cell^ might have different column sums (i.e. one cell of a particular cell type might have more RNA molecules than the other). Let $c_i,i \in \left[ {1..K} \right]$ be a sum of *i*th column of matrix **W**^cell^, i.e. coefficients *c*_*i*_ will represent RNA concentration per cell for each cell type. Let **H**^couts^ be a *K* × *M* matrix of true cell counts within each sample. Let us also note ${\mathbf{H}}^{{\mathrm{fractions}}}$ as per-sample sum-to-one normalized matrix ${\mathbf{H}}^{{\mathrm{counts}}}$:

14 $$\begin{array}{*{20}{c}} {{\mathbf{H}}_{i,j}^{{\mathrm{fractions}}} = \frac{{{\mathbf{H}}_{i,j}^{{\mathrm{counts}}}}}{{\mathop {\sum }\nolimits_{i = 1}^K {\mathbf{H}}_{i,j}^{{\mathrm{counts}}}}}.} \end{array}$$

If we know coefficients *c*_*i*_ and matrix **H**^couts^ we can easily tell how much RNA of a given cell type is in every sample, let this be matrix ${\mathbf{H}}^{{\mathrm{{rna}}}}$:

15 $$\begin{array}{*{20}{c}} {{\mathbf{C}} = \left( {\begin{array}{*{20}{c}} {c_1} & \cdots & 0 \\ \vdots & \ddots & \vdots \\ 0 & \cdots & {c_K} \end{array}} \right),{\mathbf{H}}^{{\mathrm{{rna}}}} = {\mathbf{C}} \times {\mathbf{H}}^{{\mathrm{counts}}}} \end{array}.$$

Let us also note that we can calculate ${\mathbf{H}}^{{\mathrm{rna}} - {\mathrm{fractions}}}$ in the same manner as we calculate ${\mathbf{H}}^{{\mathrm{fractions}}}$:

16 $$\begin{array}{*{20}{c}} {{\mathbf{H}}_{i,j}^{{\mathrm{rna}} - {\mathrm{fractions}}} = \frac{{{\mathbf{H}}_{i,j}^{{\mathrm{rna}}}}}{{\mathop {\sum }\nolimits_{i = 1}^K {\mathbf{H}}_{i,j}^{{\mathrm{rna}}}}}} \end{array}.$$

We will model the observed gene expression matrix **X** using an additive linear model:

${\mathbf{X}} = {\mathbf{W}}^{{\mathrm{cell}}} \times {\mathbf{H}}^{{\mathrm{counts}}}$. In usual practice, **X** matrix is normalized to account for library depth. While it can be done in several ways by calculating relative expression values like TPMs (transcripts per millions) in RNA-seq or by normalization between arrays (like quantile normalization) in microarray datasets; however, results are very similar in terms column sums: they will be equal or close to each other. We assume matrix **X** was preprocessed in the usual way, and we assume ${\mathbf{X}}^{{\mathrm{preprocessed}}}$ has equal column sums.

Let us assume ${\mathbf{X}}^{{\mathrm{preprocessed}}}$ can be fully deconvolved, i.e. we can find such ${\mathbf{W}}^{{\mathrm{dec}}},{\mathbf{H}}^{{\mathrm{dec}}}$ that

17 $$\begin{array}{*{20}{c}} {{\mathbf{X}}^{{\mathrm{preprocessed}}} = {\mathbf{W}}^{{\mathrm{dec}}} \times {\mathbf{H}}^{{\mathrm{dec}}}{\mathrm{such}}\;{\mathrm{that}}\;\forall j\mathop {\sum }\limits_{k = 1}^K {\mathbf{H}}_{k,j}^{{\mathrm{dec}}} = 1.} \end{array}$$

Since **X** was preprocessed to have the same amount of RNA within the sample, and **H** is assumed to meet a sum-to-one constraint, then **W**^dec^ is guaranteed to also have the same column sum as **X**. In this case, **H**^dec^ is nothing else but ${\mathbf{H}}^{{\mathrm{rna}} - {\mathrm{fractions}}}$.

Once we have ${\mathbf{H}}^{{\mathrm{rna}}{\mbox {-}} {\mathrm{fractions}}}$ and coefficients *ci* available we can calculate **H**^fractions^:

18 $$\begin{array}{*{20}{c}} {\frac{{c_i^{ - 1}\;{\mathbf{H}}_{i,j}^{{\mathrm{rna}}{\mbox{-}} {\mathrm{fractions}}}}}{{\mathop {\sum }\nolimits_{i = 1}^K c_i^{ - 1}\;{\mathbf{H}}_{i,j}^{{\mathrm{rna}}{\mbox{-}} {\mathrm{fractions}}}}} = \frac{{c_i^{ - 1}\frac{{{\mathbf{H}}_{i,j}^{{\mathrm{rna}}}}}{{\mathop {\sum }\nolimits_{i = 1}^K {\mathbf{H}}_{i,j}^{{\mathrm{rna}}}}}}}{{\mathop {\sum }\nolimits_{i = 1}^K c_i^{ - 1}\frac{{{\mathbf{H}}_{i,j}^{{\mathrm{rna}}}}}{{\mathop {\sum }\nolimits_{i = 1}^K {\mathbf{H}}_{i,j}^{{\mathrm{rna}}}}}}}{\mathrm{by}}\;{\mathrm{unfolding}}\;\left( {16} \right)} \\ { = \frac{{c_i^{ - 1}\;{\mathbf{H}}_{i,j}^{{\mathrm{rna}}}}}{{\mathop {\sum }\nolimits_{i = 1}^K c_i^{ - 1}{\mathbf{H}}_{i,j}^{{\mathrm{rna}}}}}{\mathrm{by}}\;{\mathrm{removing}}\mathop {\sum }\limits_{i = 1}^K {\mathbf{H}}_{i,j}^{{\mathrm{rna}}}\;\left( {{\mathrm{this}}\;{\mathrm{term}}\;{{{\rm{is}}\,{\rm{constant}}}}\;{\mathrm{for}}\;{\mathrm{every}}\;{{j}}} \right)} \\ { = \frac{{c_i^{ - 1}c_i\;{\mathbf{H}}_{i,j}^{{\mathrm{counts}}}}}{{\mathop {\sum }\nolimits_{i = 1}^K c_i^{ - 1}c_i\;{\mathbf{H}}_{i,j}^{{\mathrm{counts}}}}}{\mathrm{by}}\;{\mathrm{unfolding}}\;\left( {15} \right) = \frac{{{\mathbf{H}}_{i,j}^{{\mathrm{counts}}}}}{{\mathop {\sum }\nolimits_{i = 1}^K {\mathbf{H}}_{i,j}^{{\mathrm{counts}}}}} = {\mathbf{H}}_{i,j}^{{\mathrm{fractions}}}\;{\mathrm{by}}\;{\mathrm{folding}}\;\left( {14} \right)} \end{array}$$

### Enrichment analysis

To identify how gene sets from simplex corners were enriched in different cell subsets, we used two approaches: average *z*-score and GSEA (gene set enrichment analysis) for pairwise comparison. Log-transformed values from GSE27787, GSE45535, and DMAP were standardized for each gene (i.e. *z*-score was calculated for each gene), then for each gene set an average *z*-score across samples were calculated. For analysis of the GSE49664 dataset, differential expression analysis between erythrocytes and megakaryocytes was carried out using limma^31^ and Phantasus web-service (https://artyomovlab.wustl.edu/phantasus/): genes were ranked by the corresponding test statistics and *p* was calculated using pre-ranked gene set enrichment analysis method fgsea^32^ (https://github.com/ctlab/fgsea) package with one million gene set permutations. All heatmaps were generated using pheatmap (https://CRAN.R-project.org/package=pheatmap) package.

Cell-specific transcriptional profiles that were used for enrichment were obtained from GSE27787 for mouse hematopoietic cells, GSE49664 for primary megakaryocytes and erythroblasts from murine fetal liver hematopoietic stem/progenitor cells, GSE45535 for human blood subsets including plasma cells, and DMAP for normal human blood subsets (especially for erythrocyte contamination).

### Statistical analysis

Concordance between known and predicted cell-type proportions, between gene expression levels, between gene expression level and cell type proportions, between known and predicted gene expression levels in pure tissues was determined by Pearson correlation coefficient (*R*) or coefficient of determination (*R*^2^). Group comparisons were determined using a two-sided Mann–Whitney *U* test. All results with *p*< 0.05 were considered significant. Statistical analyses were performed with *R*.

### Reporting Summary

Further information on research design is available in the Nature Research Reporting Summary linked to this article.

## Supplementary information

Supplementary Information

Description of Additional Supplementary Files

Supplementary Data 1

Supplementary Data 2

Reporting Summary

## Data Availability

The produced RNA-seq dataset of mixed HEK and Jurkat cells is available at NCBI GEO database with accession number GSE129240.

## Code availability

All the scripts and methods proposed in this paper are available as an R-package at https://github.com/ctlab/linseed. All necessary arguments and information about removed samples to reproduce the results is present in Supplementary Table 1.
