# Supplementary material for: Complete deconvolution of cellular mixtures based on linearity of transcriptional signatures
Source: Nat Commun. 2019 May 17;10:2209. doi: 10.1038/s41467-019-09990-5 (PMC6525259; doi:10.1038/s41467-019-09990-5)
Supplement: Supplementary file 1 — Supplementary Information [file 41467_2019_9990_MOESM1_ESM.pdf]

Supplementary information

Complete deconvolution of cellular mixtures based on linearity of transcriptional signatures

Konstantin Zaitsev[1,2] et al.

[1]Department of Pathology and Immunology, Washington University School of Medicine, Saint Louis, MO, USA

[2]Computer Technologies Department, ITMO University, St. Petersburg, Russia

Supplementary note 1

Proof of correctness

Assumption of true linear model implies that

$$X = W \times H, \tag{1}$$

$$(x_{i,j}) \in \mathbb{R}_{+}^{N\times M}, (w_{i,j}) \in \mathbb{R}_{+}^{N\times K}, (h_{i,j}) \in \mathbb{R}_{+}^{K\times M}, \tag{2}$$

where N is the number of genes, K is the number of cell types and M is the number of samples, where $x_{i,j} \geq 0$ describes observed gene expression of ith gene in j^{th} sample, $w_{i,k} \geq 0$ describes gene expression of ith gene in k^{th} cell type, $h_{k,j} \geq 0$ describes proportion of kth cell type in j^{th} sample.

We also assume that $K < M \ll N$, so that

$$rank(X) = \min(rank(W), rank(H)) = \min(K, K) = K. \tag{3}$$

Gene normalization described in the paper can be written as:

$$\forall i \in [1, N], j \in [1, M] \tilde{x}_{i,j} = \frac{x_{i,j}}{\sum_{k=1}^{M} x_{i,k}} \tag{4}$$

$$\forall i \in [1, N] \tilde{X}_{i,*} = \frac{1}{\sum_{k=1}^{M} x_{i,k}} X_{i,*} (Rownormalization). \tag{5}$$

An important property of such normalization is that the sum of normalized values for any gene of the matrix equals to one:

$$\forall i \in [1, N] sum(\tilde{X}_{i,*}) = \sum_{j=1}^{M} \tilde{x}_{i,j} = \sum_{j=1}^{M} \frac{x_{i,j}}{\sum_{k=1}^{M} x_{i,k}} = \frac{\sum_{j=1}^{M} x_{i,j}}{\sum_{k=1}^{M} x_{i,k}} = 1 \tag{6}$$

Such normalization keeps non-negativity of the matrix. We can also normalize matrix H in the same manner to have matrix $\tilde{H}$ describing normalized proportions of cell types across all the samples.

In this section we prove that column vectors (genes) of matrix $\tilde{X}^T$ are lying within the $(K-1)$-simplex, formally, we must prove that there are K affinely independent vectors $u_1 \dots u_K \in \mathbb{R}_+^M$ such that for any column (gene) from $\tilde{X}^T$ there exist such coefficients $\theta_1 \dots \theta_K \geq 0$ and $\sum_{i=1}^{K} \theta_i = 1$ that any column can be presented as a sum:

$$\exists u_1 \dots u_K \in \mathbb{R}_+^M \tag{7}$$

$$\forall i \in [1, N] \exists \theta_1 \dots \theta_K \geq 0 and \sum_{k=1}^{K} \theta_k = 1 \tag{8}$$

$$\tilde{X}_{*,i}^T = \sum_{k=1}^{K} \theta_k u_k \tag{9}$$

To prove that we will show that $[u_1, u_2, \dots, u_k] = \tilde{H}^T$. Let's pick any gene i, and show that $\tilde{x}_{*,i}^T$ can be described as sum of columns of $\tilde{H}^T$.

In case of linear model, it is true that $X_{i,*} = \sum_{j=1}^{K} w_{i,j} H_{j,*}$ we can rewrite this equation in terms of $\tilde{X}$ and $\tilde{H}$:

$$\tilde{X}_{i,*} = \frac{\sum_{j=1}^{K} w_{i,j} H_{j,*}}{\sum_{k=1}^{M} x_{i,k}} \tag{10}$$

$$\tilde{X}_{i,*} = \frac{\sum_{j=1}^{K} w_{i,j} \left(\sum_{l=1}^{M} h_{j,l}\right) \tilde{H}_{j,*}}{\sum_{k=1}^{M} x_{i,k}} \tag{11}$$

$$\tilde{X}_{i,*} = \sum_{j=1}^{K} \frac{w_{i,j} \left(\sum_{l=1}^{M} h_{j,l}\right)}{\sum_{k=1}^{M} x_{i,k}} \tilde{H}_{j,*} \tag{12}$$

Let $\frac{w_{i,j} (\sum_{l=1}^{M} h_{j,l})}{\sum_{k=1}^{M} x_{i,k}}$ be $\alpha_{i,j}$ and let's now prove that

$$\tilde{X}_{i,*} = \sum_{j=1}^{K} \alpha_{i,j} \tilde{H}_{j,*} \Rightarrow \sum_{j=1}^{K} \alpha_{i,j} = 1 \tag{13}$$

To prove that we will use the property of our normalization: sums of elements of vectors $\tilde{X}_{i,*}$ and $\tilde{H}_{i,*}$ are equal to one and since matrices $\tilde{X}$ and $\tilde{H}$ are non-negative we can write that:

$$1 = sum\left(\tilde{X}_{i,*}\right) = \sum_{j=1}^{K} \alpha_{i,j} sum\left(\tilde{H}_{j,*}\right) = \sum_{j=1}^{K} \alpha_{i,j} \tag{14}$$

where sum denotes sum of the vector elements. Now we can just transpose the vectors $\tilde{x}_{i,*}$ and $\tilde{h}_{j,*}$ and see that any column of $\tilde{X}^T$ can be written as weighted sums of columns of $\widetilde{H}^T$ where sum of weights equals one, hence every column vector of $\tilde{X}^T$ lies within $(K-1)$-simplex generated by column vectors of $\widetilde{H}^T$.

$$\tilde{X}^T_{*,i} = \sum_{j=1}^{K} \alpha_{i,j} \widetilde{H}^T_{*,j} \ and \ \sum_{j=1}^{K} \alpha_{i,j} = 1 \qquad (15)$$

In case of true linear model, normalization puts all the genes on some simplex and simplex corners are defined by the proportions of the cell types.

Only thing left to mention that after normalization signature genes will be in the corners of such simplex. We call gene i signature gene if it has only one non-zero value in $w_{i,*}$:

$$gene\ i\ is\ signature \Leftrightarrow \exists!\, j \in [1, K]\ w_{i,j} \neq 0 \qquad (16)$$

Let's assume gene i is signature for cell type j. Then due to linear model:

$$X_{i,*} = w_{i,j}\ H_{j,*} \qquad (17)$$

we can rewrite it in terms of in terms of $\tilde{X}$ and $\widetilde{H}$:

$$\tilde{X}_{i,*} = \frac{w_{i,j}\ H_{j,*}}{sum(X_{i,*})} = \frac{w_{i,j}\ H_{j,*}}{sum(w_{i,j}\ H_{j,*})} = \frac{w_{i,j}\ H_{j,*}}{w_{i,j}\ sum(H_{j,*})} = \frac{H_{j,*}}{sum(H_{j,*})} = \widetilde{H}_{j,*} \qquad (18)$$

$$\tilde{X}^T_{*,i} = \widetilde{H}^T_{*,j} \qquad (19)$$

Hence any true signature gene will be in the corresponding corner of such simplex.

Supplementary figure 1

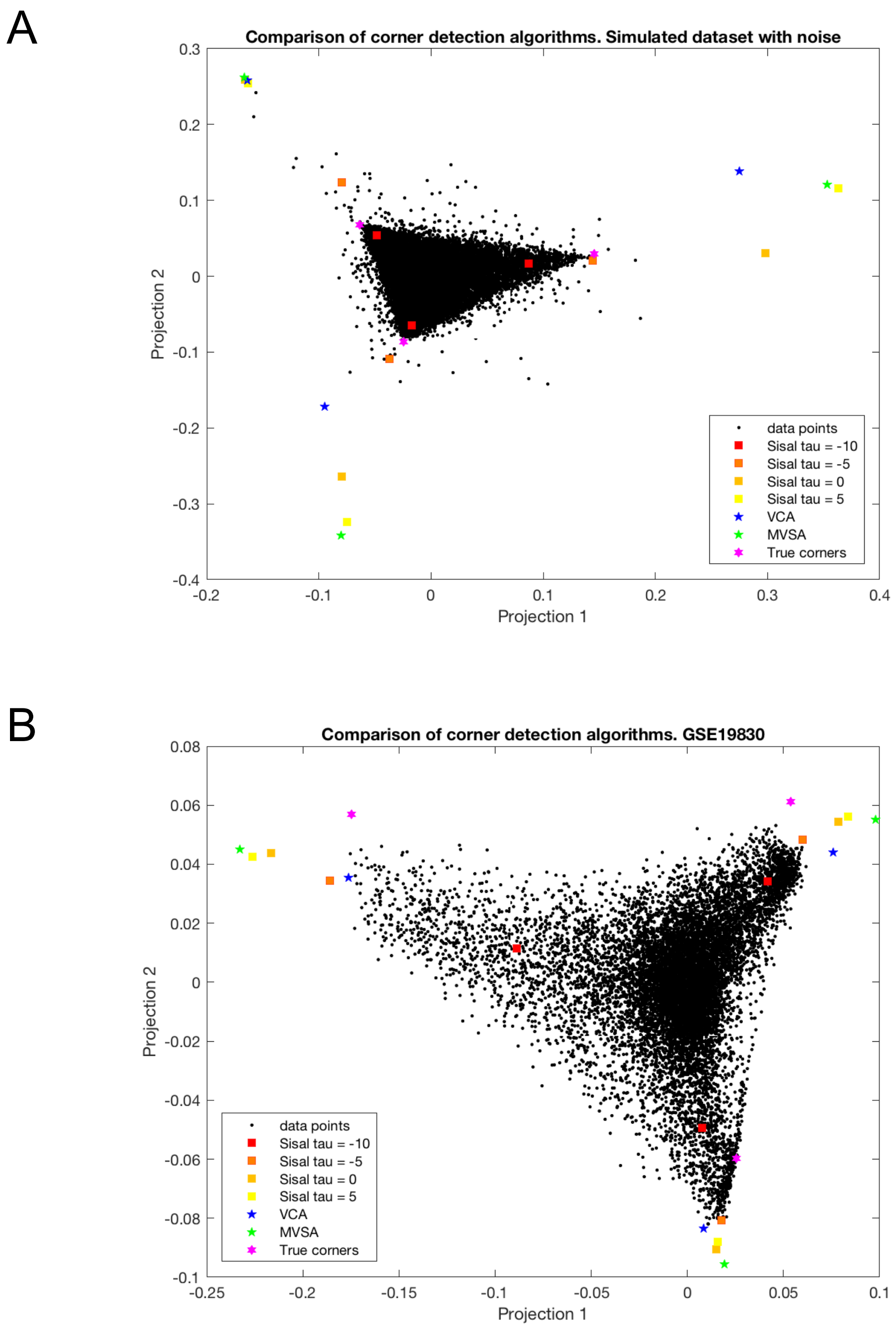

Supplementary figure 1. Comparative analysis of geometric simplex identification algorithms
We applied three algorithms (MVSA, VCA, and SISAL) for geometric simplex corner identification to **(a)** simulated data with noise, or to **(b)** benchmark dataset GSE19830. VCA finds the best simplex corners from points of your dataset, MVSA finds simplex of minimum volume that encloses all the points and SISAL tries to find simplex of minimum volume with a penalty for points outside of the simplex. SISAL taus on the panels are log2 values, actual taus are: 2-10, 2-5, 20, 25.

Supplementary figure 2

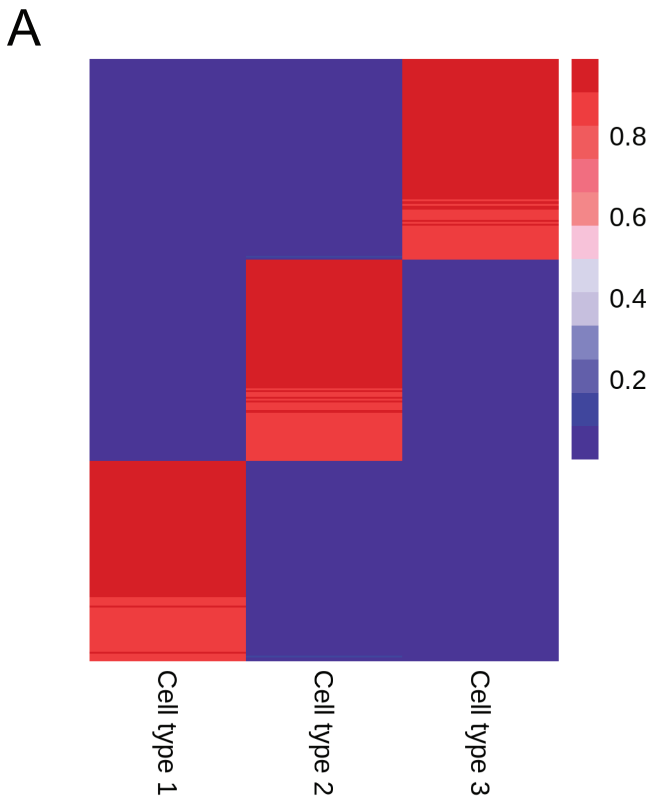

Supplementary figure 2. Expression of markers identified in Fig 4a
a. The heatmap shows cell-specific expression of identified corner genes in simulated pure cell types, the matrix of pure gene expression is row-normalized to have the sum of one.

Supplementary figure 3

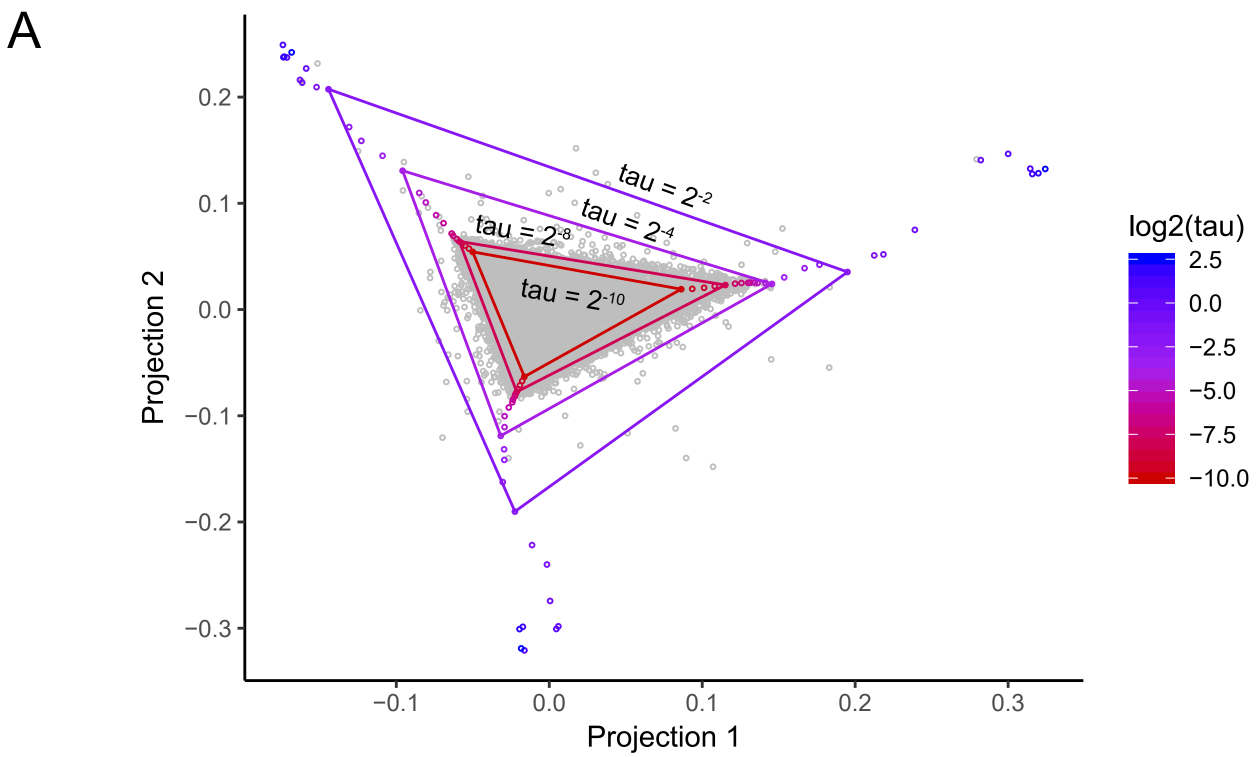

Supplementary figure 3. Illustration of simplex volume for different tolerance parameter (tau)
a. Simulated dataset with noise was projected onto linear subspace and simplex corners were identified with SISAL with different taus. Smaller taus generate simplexes of smaller volume while simplexes with large taus enclose more points.

Supplementary figure 4

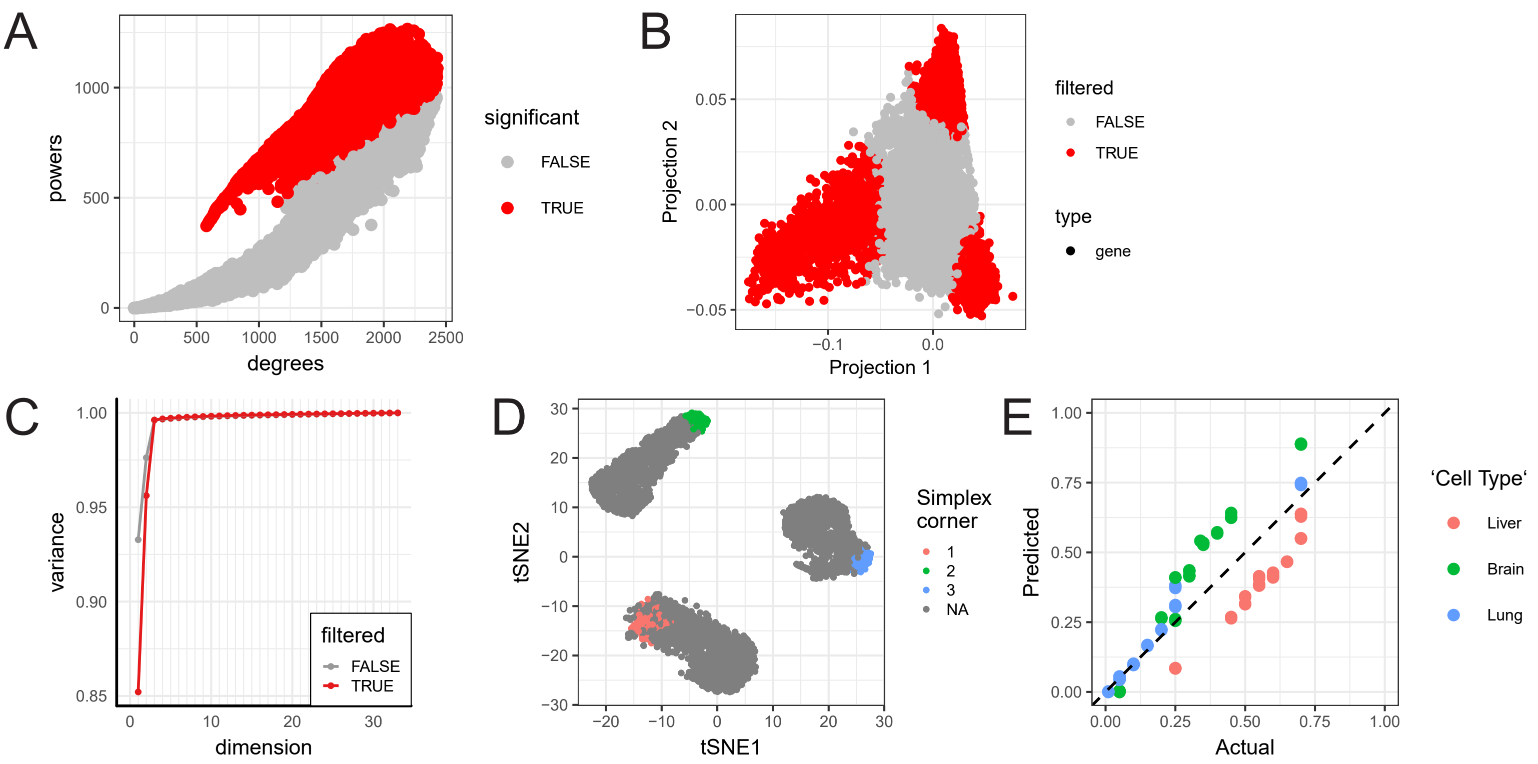

Supplementary figure 4: the result of Linseed pipeline applied to benchmark dataset GSE19830
a. Illustration of filtering procedure applied to the dataset. **b.** Projection shows which genes left after the filtering procedure. **c.** Variance explained by each dimension of SVD, 3 components can be easily seen. **d.** TSNE applied to genes left after the filtering. **e.** Comparison of predicted proportions to actual proportions of the cell type in mixed samples.

Supplementary figure 5

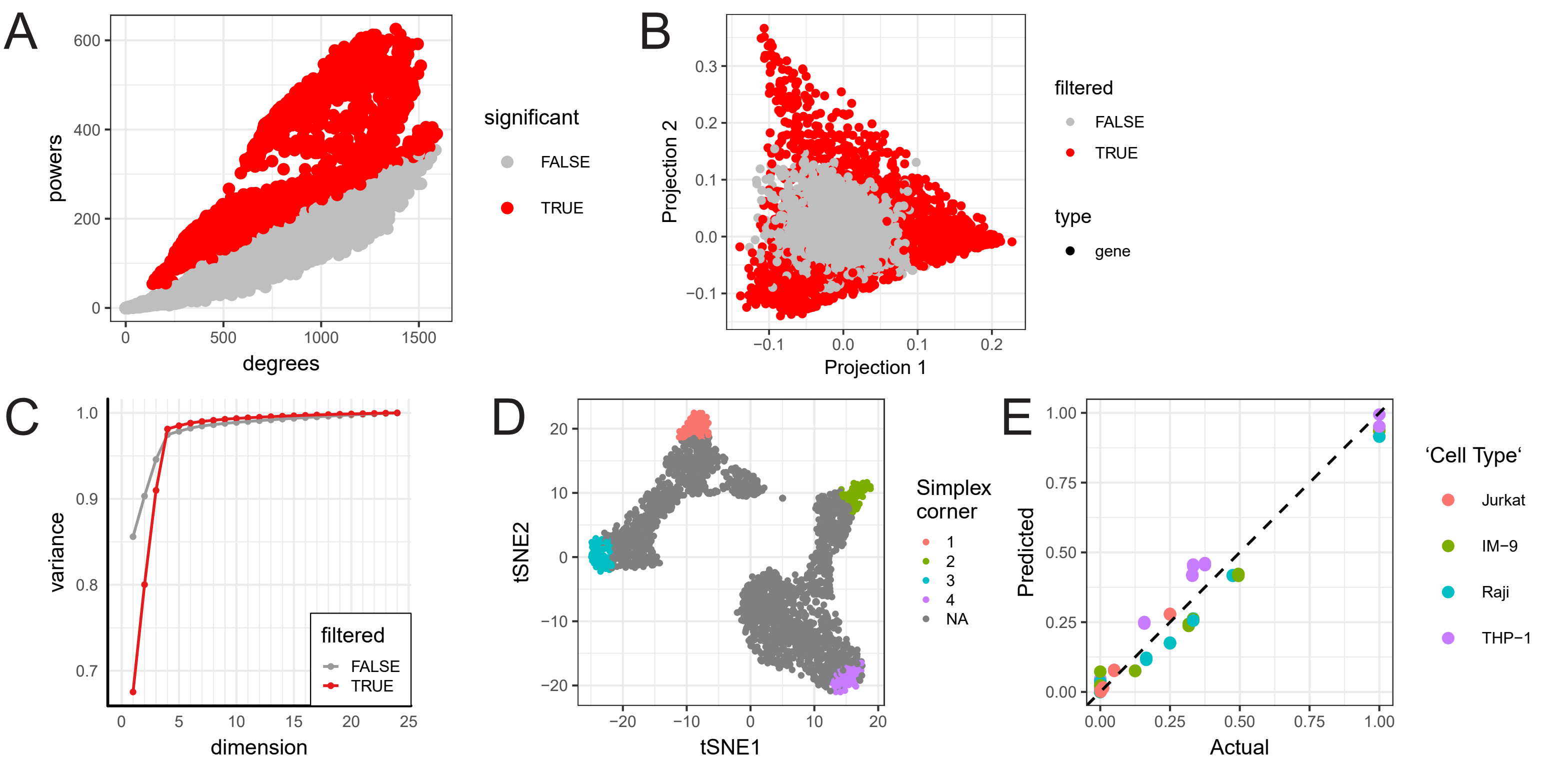

Supplementary figure 5: the result of Linseed pipeline applied to benchmark dataset GSE11058
a. Illustration of filtering procedure applied to the dataset. **b.** Projection shows which genes left after the filtering procedure.
c. Variance explained by each dimension of SVD, 3 components can be easily seen. **d.** TSNE applied to genes left after filtering.
e. Comparison of predicted proportions to actual proportions of the cell type in mixed samples.

Supplementary figure 6

A

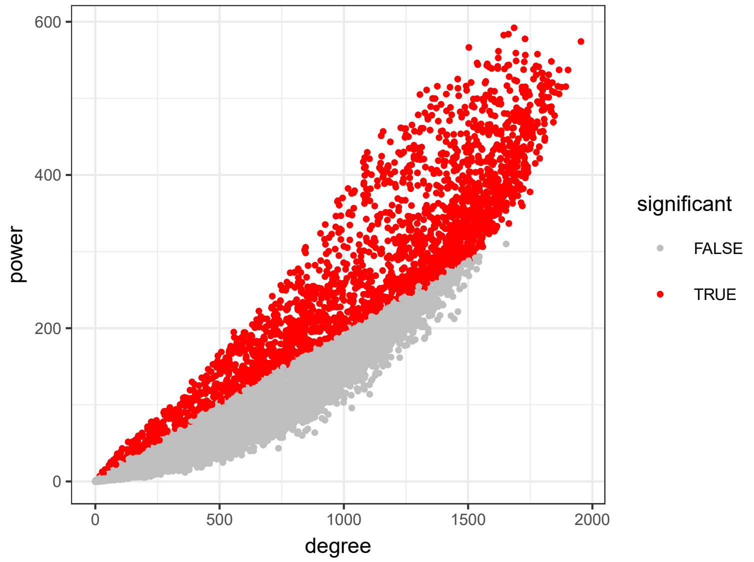

Supplementary figure 6: filtering procedure for mouse dataset GSE27563
a. Illustration of filtering procedure applied to the mouse dataset GSE27563.

Supplementary figure 7

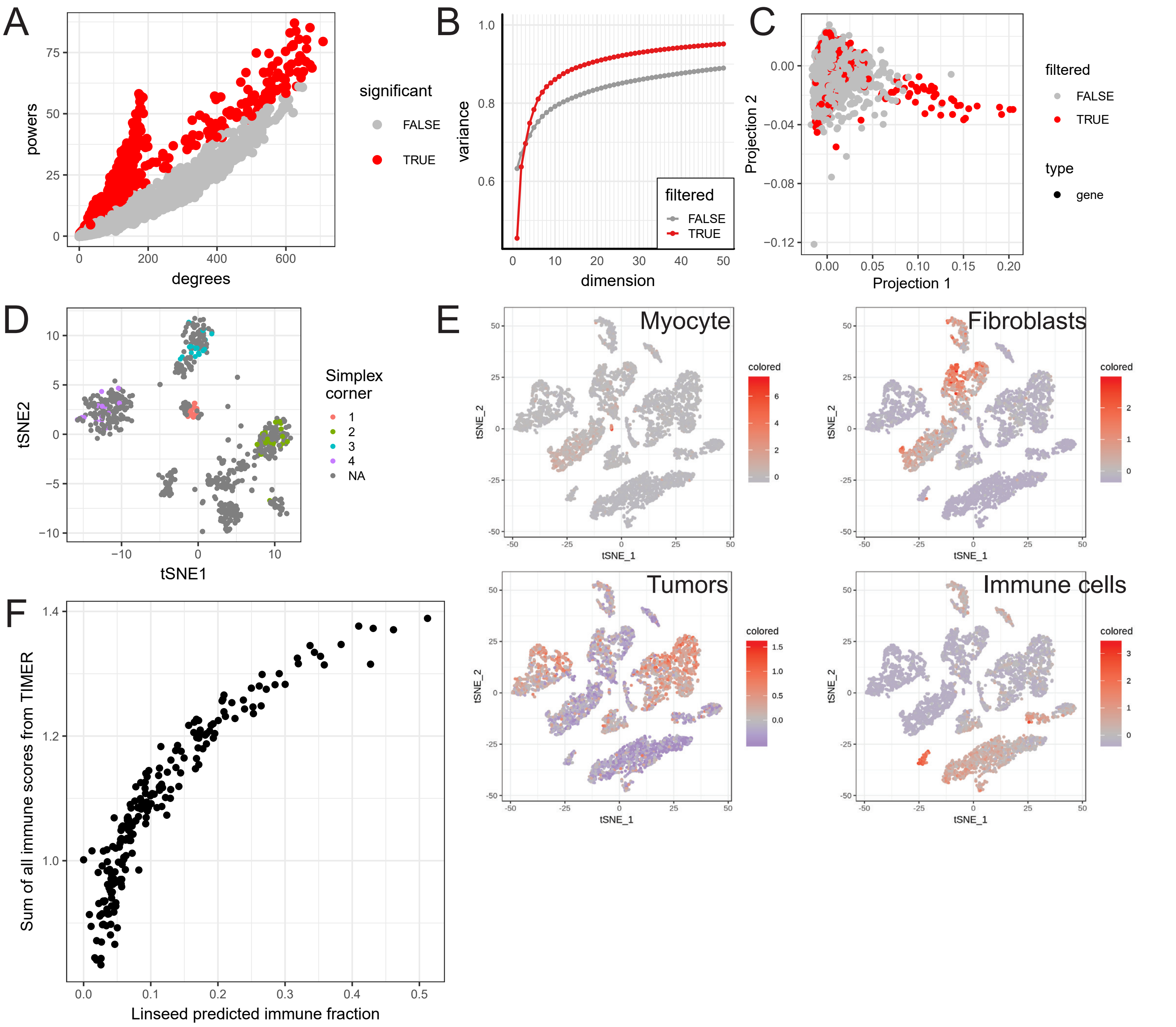

Supplementary figure 7: the result of Linseed pipeline applied to TCGA HNSCC dataset
a. Illustration of filtering procedure applied to the dataset. **b.** Variance explained by each dimension of SVD. **c.** Projection shows which genes left after the filtering procedure, 3 components can be easily seen. **d.** TSNE applied to genes left after the filtering. **e.** Closest 100 genes to each corner were used to generate averages expression for single-cell HNSCC dataset GSE103322. These average expression profiles highlight different cellular populations within the tumor samples. **f.** Comparison predicted proportions of immune cell types in the mixture against the sum of the immune scores predicted by TIMER.

Supplementary figure 8

A

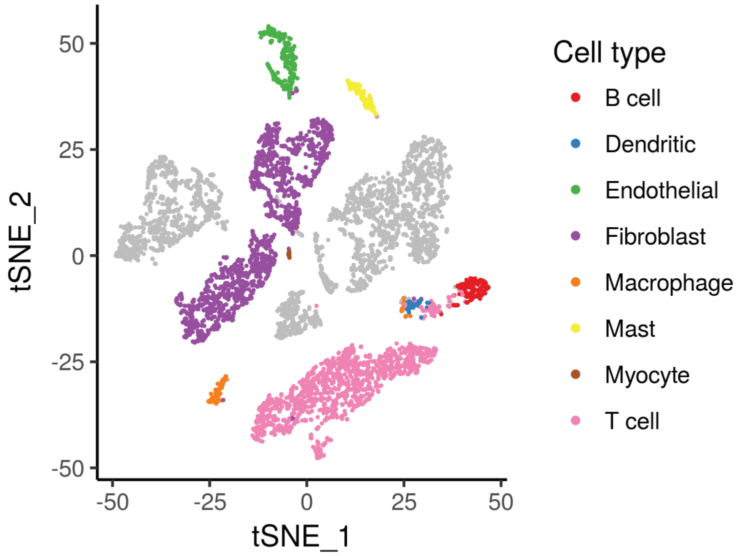

B

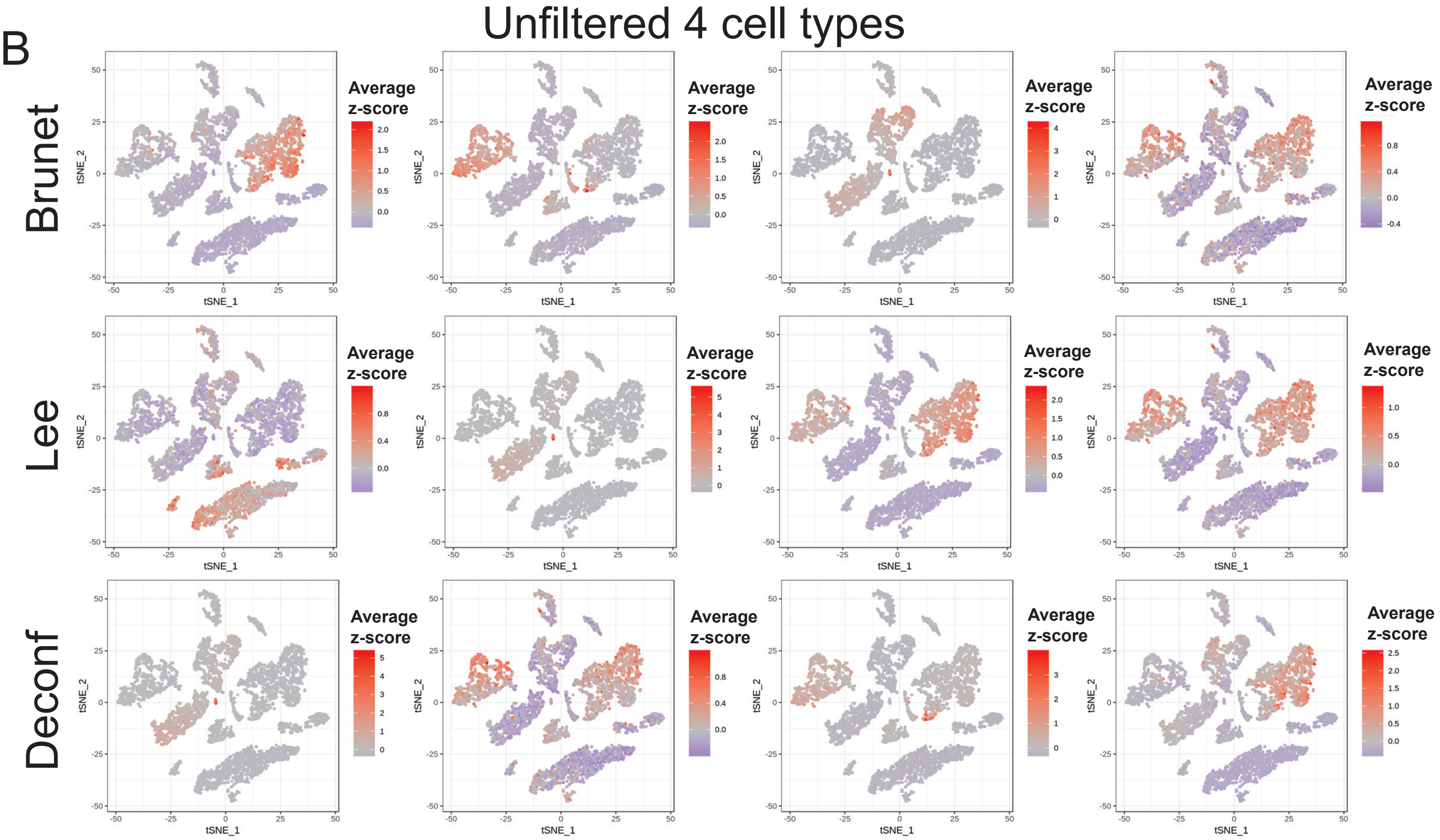

C

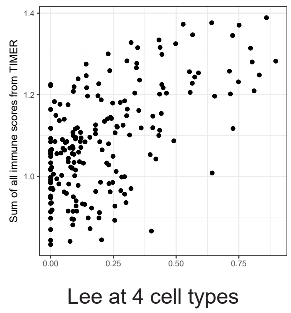

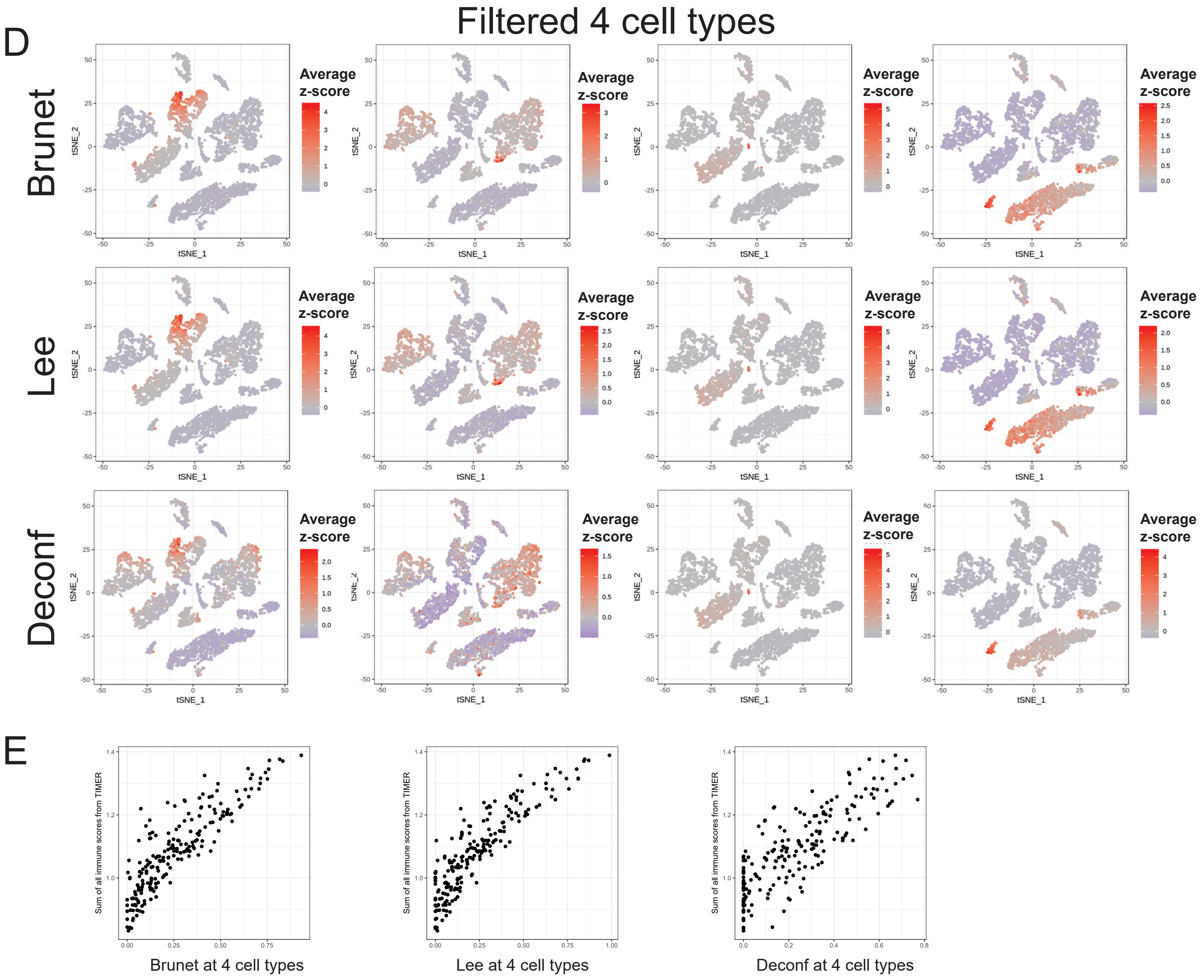

Supplementary figure 8: application of NMF methods to HNSCC TCGA dataset
We evaluated three different NMF algorithms for their ability to identify immune cell populations in HNSCC datasets: deconf complete deconvolution algorithm and two NMF approaches proposed by Lee et al. and Brunet et al. We run all these methods for both unfiltered and filtered datasets. After deconvolution we took 50 top markers for each gene set based on expression in signature matrix W. **a.** Public scRNA-seq dataset of HNSCC (Puram et al, 2017) with cell type annotations: clusters of interest are immune cells at the bottom. On panels **(b)** and **(d)** we show how these gene sets enrich publicly available scRNA-seq dataset of HNSCC. **b.** Enrichment plots of signature genes identified by three approaches when unfiltered dataset used as an input. Brunet and Deconf did not identify immune signatures in TNSCC TCG. Lee identified immune signatures (first panel in lee algorithm). **c.** Comparison of TIMER scores and immune proportions predicted by Lee method. **d.** Enrichment plots of signature genes identified by three approaches when filtered dataset used as an input. All three methods identified gene set related to immune signatures (last panel for every algorithm). **e.** Comparison of TIMER scores and immune proportions predicted by all three methods. It is evident that filtering procedure enabled these algorithms to accurate prediction of immune proportions when compared with TIMER scores.

Supplementary figure 9

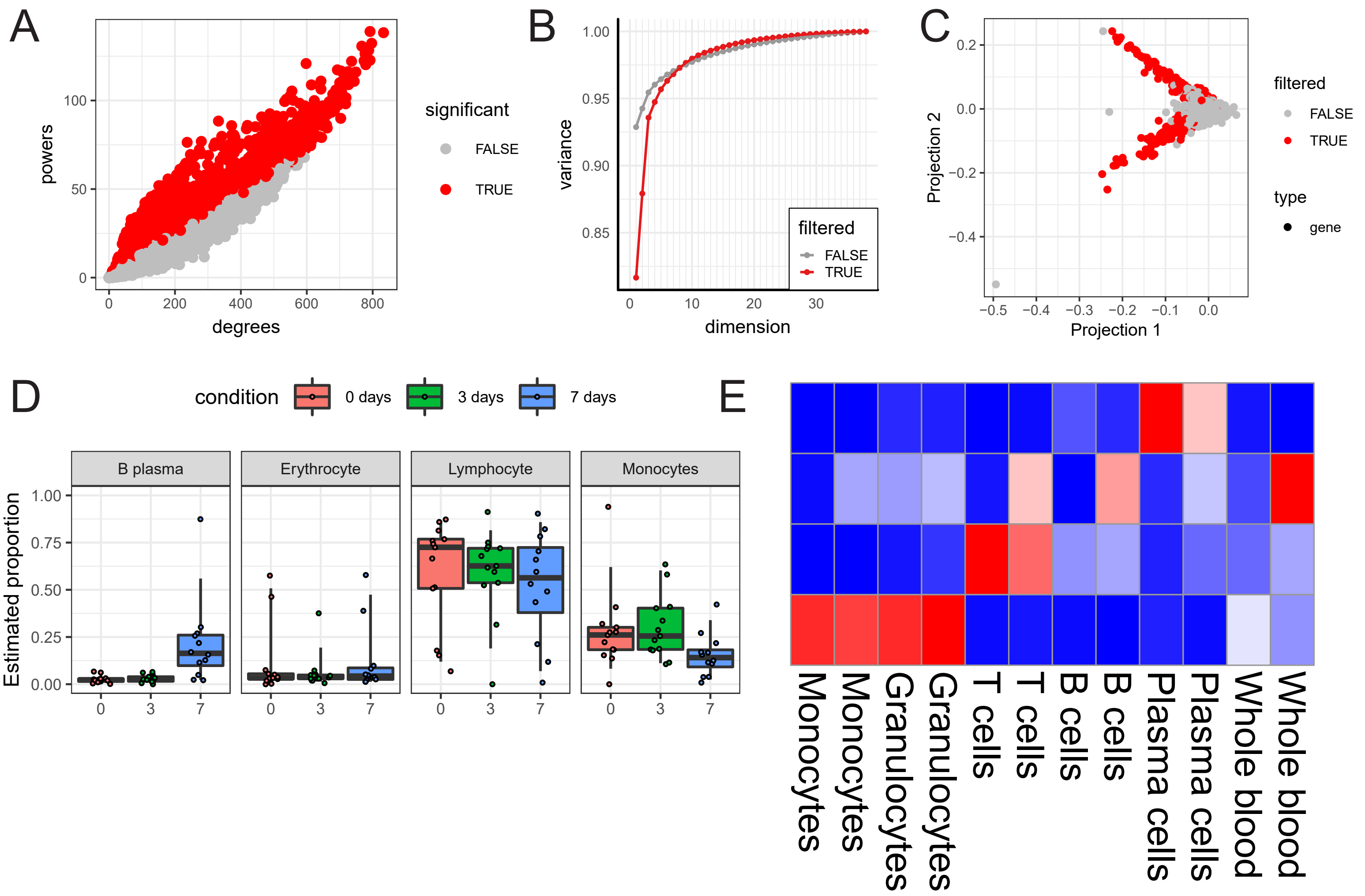

Supplementary figure 9: the result of Linseed pipeline applied to MCV4 vaccination dataset (GSE52245)
a. Illustration of filtering procedure applied to the dataset. **b.** Variance explained by each dimension of SVD. **c.** Projection shows which genes left after the filtering procedure, 3 components can be easily seen. **d.** Box plots showing predicted proportions for 0, 3 and 7 days post-vaccination. For each boxplot bottom whisker, bottom of the box, middle line, top of the box and top whisker represent 5%, 25%, 50% (median), 75% and 95% quantiles. **e.** Closest 100 genes to each corner were used to generate averages expression for GSE45535 to identify cell types in the mixture.

Supplementary figure 10

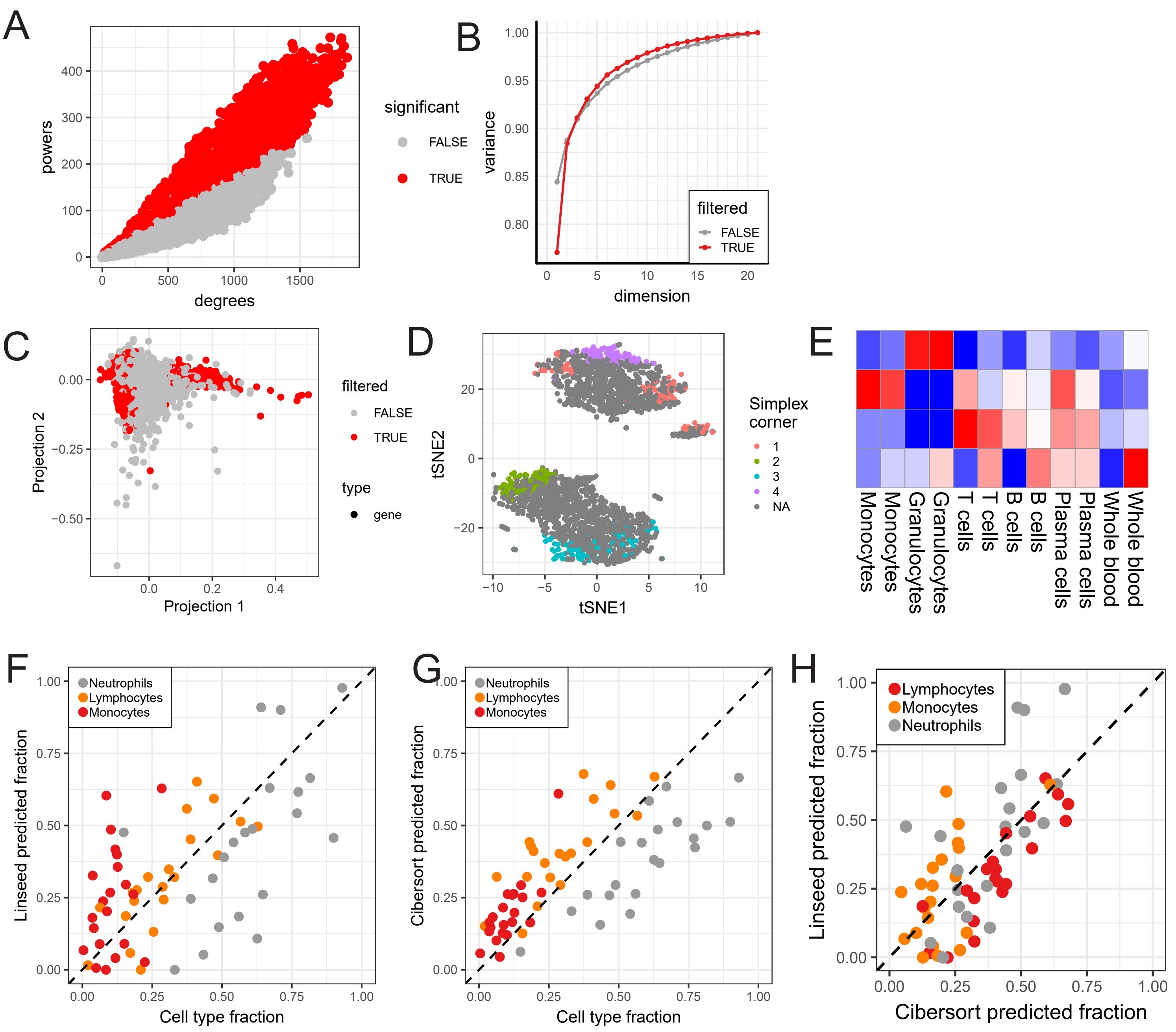

Supplementary figure 10: the result of Linseed pipeline applied to GSE20300 – whole blood gene expression analysis of stable and acute rejection pediatric kidney transplant patients

a. Illustration of filtering procedure applied to the dataset. **b.** Variance explained by each dimension of SVD. **c.** Projection shows which genes left after filtering procedure, 3 components can be easily seen. **d.** TSNE applied to genes left after. **e.** Closest 100 genes to each corner were used to generate averages expression for GSE45535 to identify cell types in the mixture. **f.** Comparison of LinSeed predicted fraction to actual blood counts. **g.** Comparison of CIBERSORT predicted fractions to actual blood counts. **h.** Comparison of CIBERSORT predicted fractions to LinSeed predicted fractions.

Supplementary figure 11

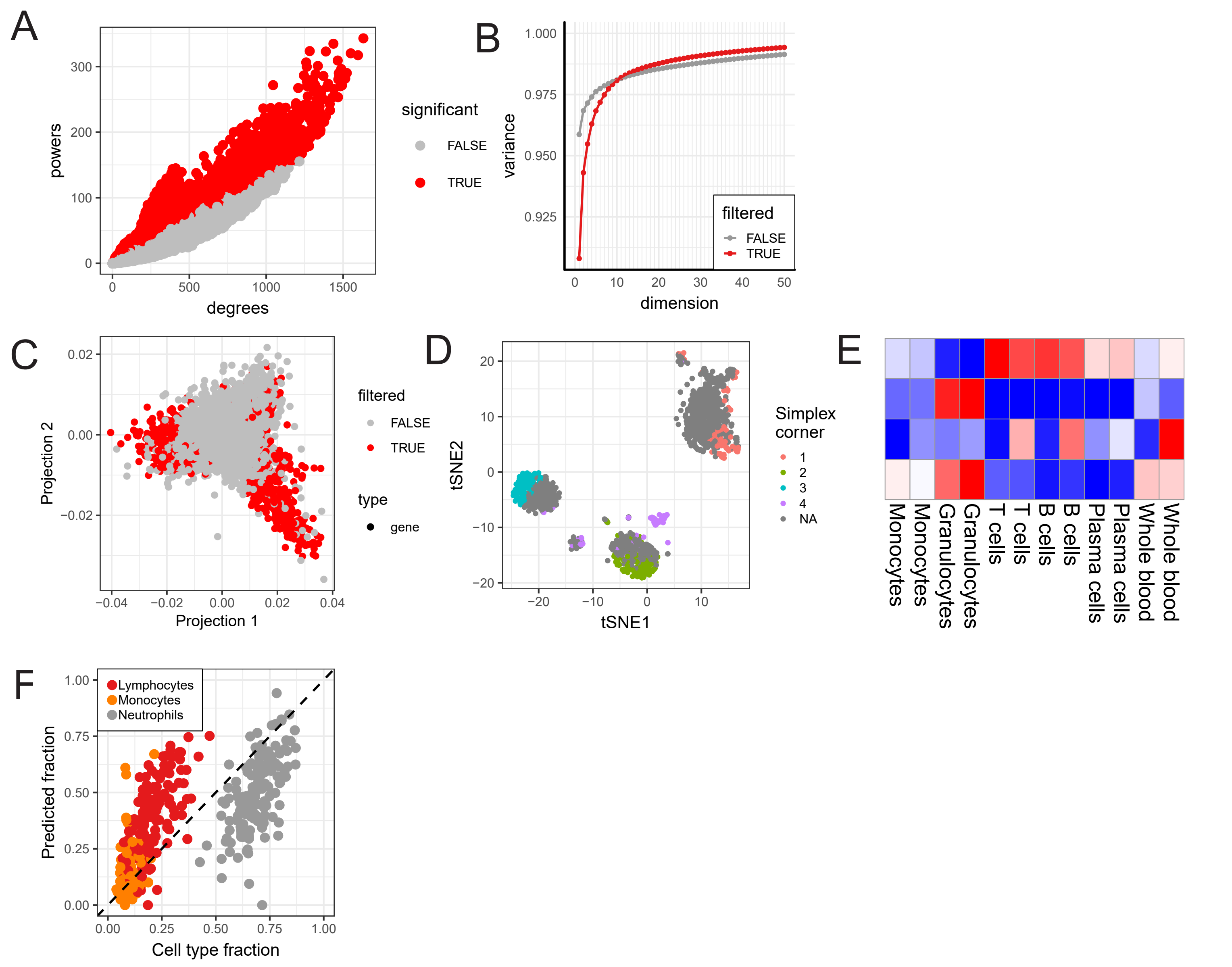

Supplementary figure 11: the result of Linseed pipeline applied to GSE77343 – whole blood gene expression in chronic heart failures

a. Illustration of filtering procedure applied to the dataset. **b.** Variance explained by each dimension of SVD. **c.** Projection shows which genes left after filtering procedure, 3 components can be easily seen. **d.** TSNE applied to genes left after. **e.** Closest 100 genes to each corner were used to generate averages expression for GSE45535 to identify cell types in the mixture. **f.** Comparison of LinSeed predicted fraction to actual blood counts.

Supplementary figure 12

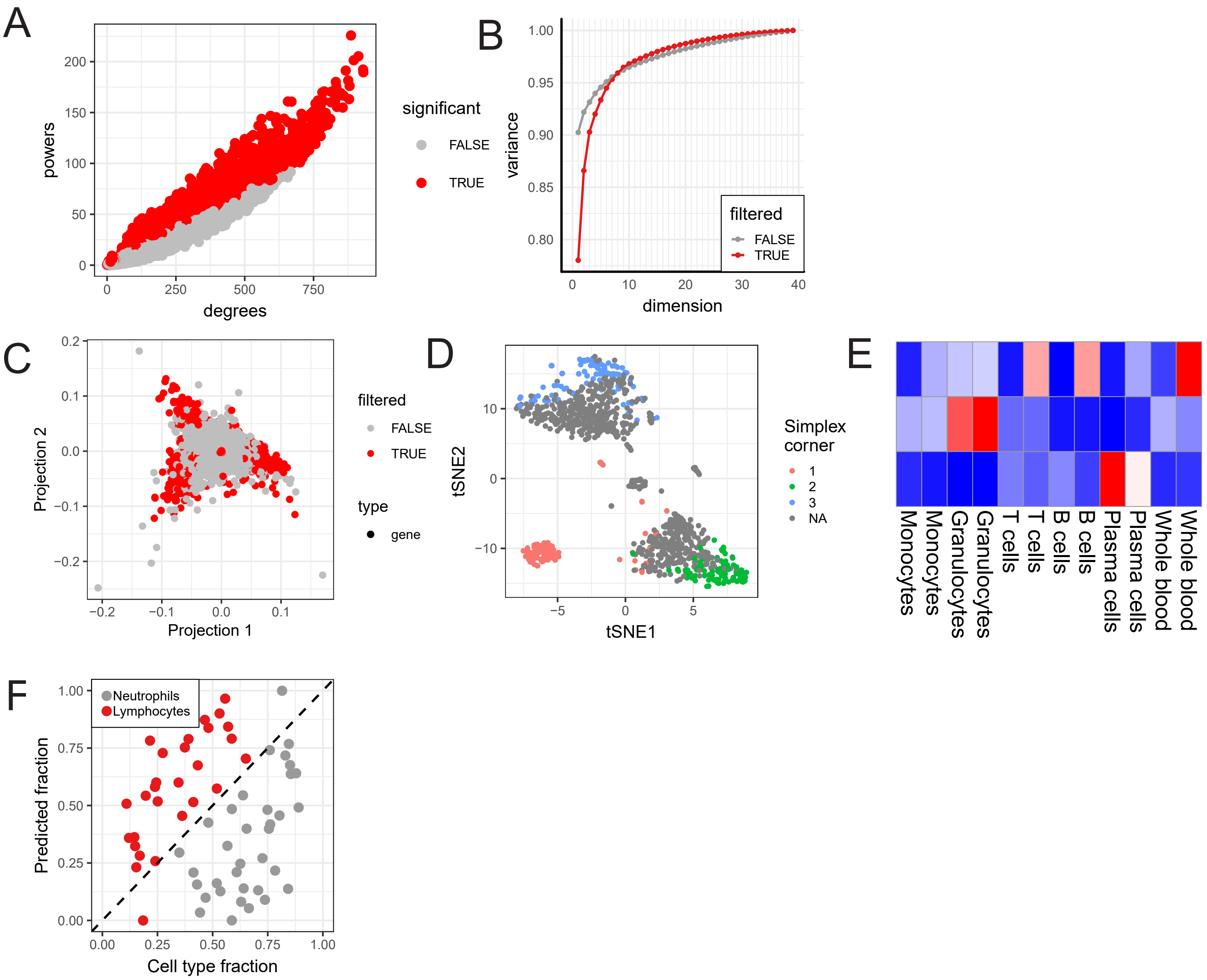

Supplementary figure 12: the result of Linseed pipeline applied to E-MTAB-6413
a. Illustration of filtering procedure applied to the dataset. **b.** Variance explained by each dimension of SVD. **c.** Projection shows which genes left after the filtering procedure, 3 components can be easily seen. **d.** TSNE applied to genes left after. **e.** Closest 100 genes to each corner were used to generate averages expression for GSE45535 to identify cell types in the mixture. **f.** Comparison of LinSeed predicted fraction to actual blood counts.

Supplementary figure 13

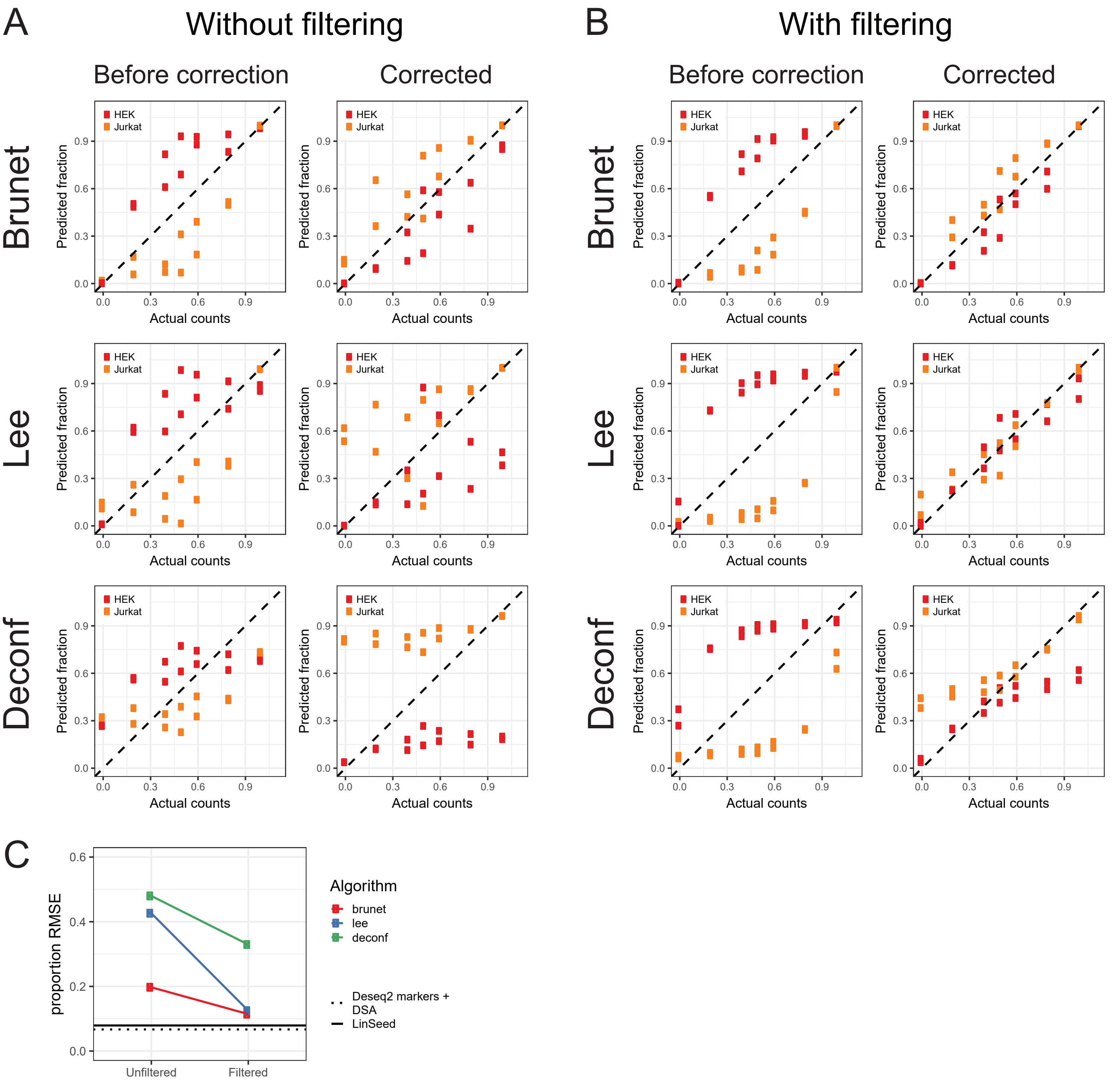

Supplementary figure 13: application of NMF methods to HEK/Jurkat mixture dataset
We evaluated *deconf* complete deconvolution method and two NMF approached proposed by *Lee* et al. and *Brunet* et al. We applied these methods to the mixtures of HEK and Jurkat cell lines without **(a)** and with **(b)** the filtering procedure. Each panel shows proportions before and after correction for each of the methods. Filtering improves the performance of all methods.
c. All algorithms were run 10 times with different initial random seed, points showing mean RMSE of 10 runs. Filtering procedure improves mean RMSE of all methods.

Supplementary figure 14

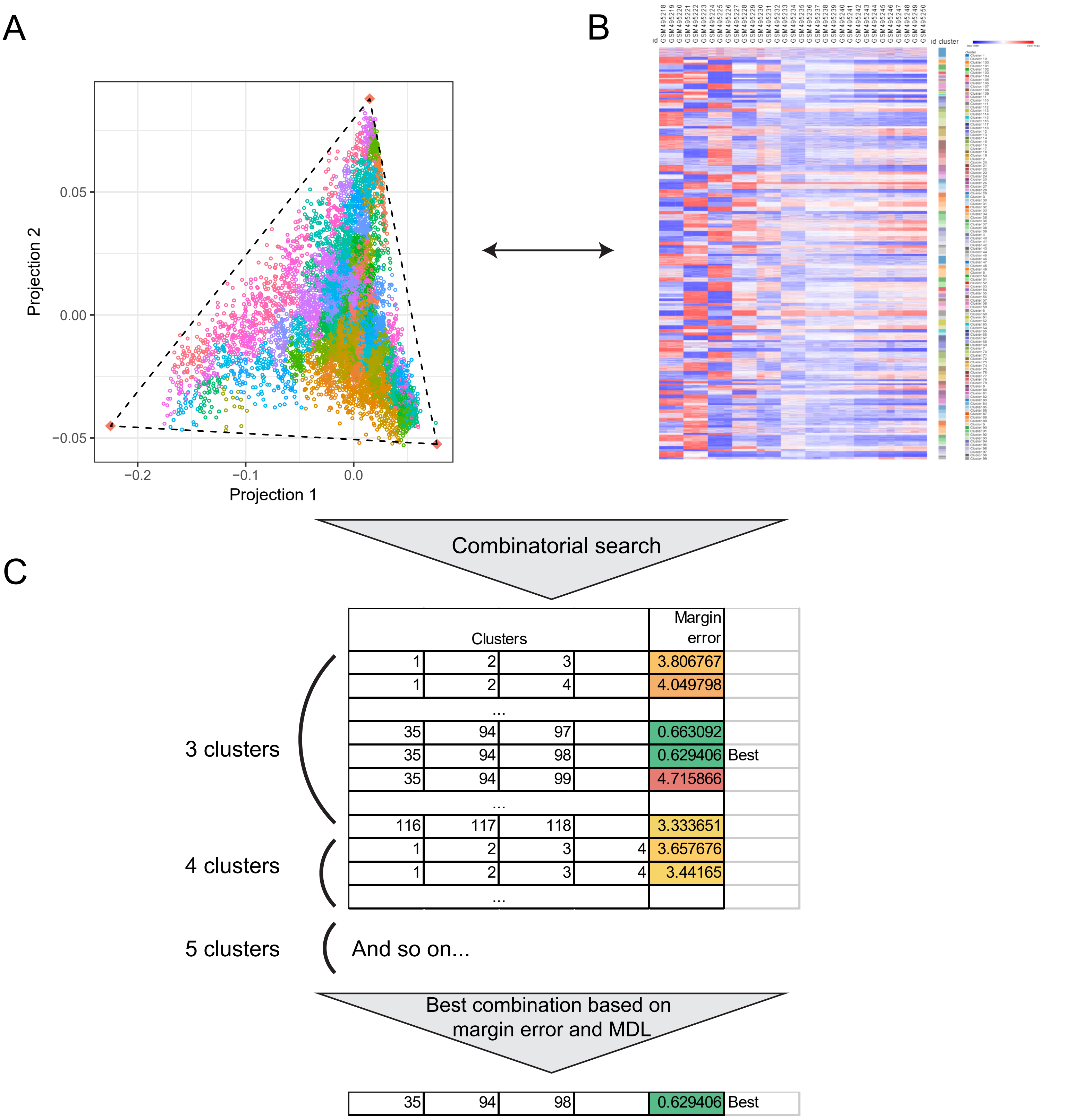

Supplementary figure 14. An illustration of the approach used by Wang et al Sci Rep (2016).
a. Whole gene space is clustered using affinity propagation. **b.** This is equivalent to clustering gene expression based on similar expression patterns: heatmap shows expression of gene clusters from **(a)**. **c.** An algorithm implemented in Wang et al then evaluates all combination of the clusters from (**a**,**b**) for the ability to reconstruct input gene expression matrix (for each set of cluster centroids convex-hull-to-data fitting criterion is evaluated by margin-of-error). Then the best combination is chosen and considered to be deconvolution.

Supplementary table 1

Dataset	Samples description	Number of samples	Number of genes	Cell type number
Simulation dataset	All 40 samples	40	12000	3
Simulation dataset with noise	All 40 samples	40	12000	3
GSE19830	Mixed samples only: from GSM495218 to GSM495250	33	12000	3
GSE11058	All samples	24	12000	4
GSE27563	All samples from training set, and sample GSM682181 removed as an outlier	45	12000	5
GSE20300	Samples GSM508894, GSM508903, GSM508906 removed as outliers	21	12000	4
GSE52245	MCV4 vaccination, 13 female donors, GSM1261788 was removed as outlier	38	12000	4
GSE77343	All male samples from the dataset. Samples GSM2049539, GSM2049557, GSM2049467, GSM204959, GSM2049604, GSM2049608,GSM 2049652 were considered outliers	142		4
Gambian kids	All samples	39	12000	3
TCGA dataset	All male samples	415	10000	4
HEK/Jurkat mixture	All samples	14	12000	2

Supplementary table 1. Arguments and parameters used in the study
Table describes all the parameters that are used in the study. Table also informs about samples that were removed from the datasets prior the analysis.
