# Supplementary material for: Complete deconvolution of cellular mixtures based on linearity of transcriptional signatures
Source: Nat Commun. 2019 May 17;10:2209. doi: 10.1038/s41467-019-09990-5 (PMC6525259; doi:10.1038/s41467-019-09990-5)
Supplement: Supplementary file 2 — Description of Additional Supplementary Files [file 41467_2019_9990_MOESM2_ESM.pdf]

Description of Additional Supplementary Files

File Name: Supplementary Data 1
Description: 3 gene sets of mutually linear genes from analysis from Fig. 1.

File Name: Supplementary Data 2
Description: Collinearity network of TCGA HNSCC dataset presented in Fig. 2.
