# Supplementary material for: Complete deconvolution of cellular mixtures based on linearity of transcriptional signatures
Source: Nat Commun. 2019 May 17;10:2209. doi: 10.1038/s41467-019-09990-5 (PMC6525259; doi:10.1038/s41467-019-09990-5)
Supplement: Supplementary file 5 — Reporting Summary [file 41467_2019_9990_MOESM5_ESM.pdf]

nature research

Corresponding author(s): Maxim N. Artyomov

Last updated by author(s): Apr 4, 2019

Reporting Summary

Nature Research wishes to improve the reproducibility of the work that we publish. This form provides structure for consistency and transparency in reporting. For further information on Nature Research policies, see Authors & Referees and the Editorial Policy Checklist.

Statistics

For all statistical analyses, confirm that the following items are present in the figure legend, table legend, main text, or Methods section.

n/a	Confirmed	
☒	☐	The exact sample size (*n*) for each experimental group/condition, given as a discrete number and unit of measurement
☒	☐	A statement on whether measurements were taken from distinct samples or whether the same sample was measured repeatedly
☐	☒	The statistical test(s) used AND whether they are one- or two-sided<br>*Only common tests should be described solely by name; describe more complex techniques in the Methods section.*
☒	☐	A description of all covariates tested
☒	☐	A description of any assumptions or corrections, such as tests of normality and adjustment for multiple comparisons
☒	☐	A full description of the statistical parameters including central tendency (e.g. means) or other basic estimates (e.g. regression coefficient) AND variation (e.g. standard deviation) or associated estimates of uncertainty (e.g. confidence intervals)
☐	☒	For null hypothesis testing, the test statistic (e.g. *F*, *t*, *r*) with confidence intervals, effect sizes, degrees of freedom and *P* value noted<br>*Give P values as exact values whenever suitable.*
☒	☐	For Bayesian analysis, information on the choice of priors and Markov chain Monte Carlo settings
☒	☐	For hierarchical and complex designs, identification of the appropriate level for tests and full reporting of outcomes
☒	☐	Estimates of effect sizes (e.g. Cohen's *d*, Pearson's *r*), indicating how they were calculated

Our web collection on statistics for biologists contains articles on many of the points above.

Software and code

Policy information about availability of computer code

Data collection	Demultiplexed single-end fastq files were aligned to the mixture reference GRCh38 and ERCC spike-in sequences by top-level assembly with STAR (version 2.6.1b). Gene counts were produced RSEM (version v1.3.1).
Data analysis	All the scripts and methods proposed in this paper are available as an R-package at https://github.com/ctlab/linseed.

For manuscripts utilizing custom algorithms or software that are central to the research but not yet described in published literature, software must be made available to editors/reviewers. We strongly encourage code deposition in a community repository (e.g. GitHub). See the Nature Research guidelines for submitting code & software for further information.

Data

Policy information about availability of data

All manuscripts must include a data availability statement. This statement should provide the following information, where applicable:

- Accession codes, unique identifiers, or web links for publicly available datasets
- A list of figures that have associated raw data
- A description of any restrictions on data availability

The produced RNA-seq dataset of mixed HEK and Jurkat cells is available at NCBI GEO database with accession number GSE129240.

Field-specific reporting

Please select the one below that is the best fit for your research. If you are not sure, read the appropriate sections before making your selection.

☒ Life sciences ☐ Behavioural & social sciences ☐ Ecological, evolutionary & environmental sciences

Life sciences study design

All studies must disclose on these points even when the disclosure is negative.

Sample size	For mixtures of HEK and Jurkat cells we have two samples for each proportions: 7 different proportions, 14 samples at total. Rationale behind this is to have enough samples to run complete deconvolution algorithms.
Data exclusions	In public dataset we removed samples that are either outliers or samples to keep remaining samples sex-matched. Complete list of samples removed from the study is available at Supplementary table 3. No samples were removed from generated HEK/Jurkat dataset.
Replication	NA
Randomization	NA
Blinding	NA

Reporting for specific materials, systems and methods

We require information from authors about some types of materials, experimental systems and methods used in many studies. Here, indicate whether each material, system or method listed is relevant to your study. If you are not sure if a list item applies to your research, read the appropriate section before selecting a response.

Materials & experimental systems

n/a	Involved in the study
☒	☐ Antibodies
☐	☐ Eukaryotic cell lines
☒	☐ Palaeontology
☒	☐ Animals and other organisms
☒	☐ Human research participants
☒	☐ Clinical data

Methods

n/a	Involved in the study
☒	☐ ChIP-seq
☒	☐ Flow cytometry
☒	☐ MRI-based neuroimaging

Eukaryotic cell lines

Policy information about cell lines

Cell line source(s)	HEK-293T from ATCC (ATCC CRL-321666), Jurkat cells provided by Prof. Schreiber laboratory (Department of Pathology and Immunology, Washington University School of Medicine, USA)
Authentication	HEK-293T from ATCC authenticated for STR DNA profiling by ATCC, no further authentication performed in laboratory.
Mycoplasma contamination	Not tested. Authentic cell culture samples are stored for eventual mycoplasma test.
Commonly misidentified lines (See ICLAC register)	No commonly misidentified lines were used.
